# Molecular epidemiology of continued *Plasmodium falciparum* disease transmission after an outbreak in Ecuador

**DOI:** 10.3389/fitd.2023.1085862

**Published:** 2023-03-16

**Authors:** Shazia Ruybal-Pesántez, Fabián E. Sáenz, Samantha L. Deed, Erik K. Johnson, Daniel B. Larremore, Claudia A. Vera-Arias, Kathryn E. Tiedje, Karen P. Day

**Affiliations:** 1 School of BioSciences/Bio21 Institute, The University of Melbourne, Melbourne, VIC, Australia,; 2 Centro de Investigación para la Salud en América Latina, Facultad de Ciencias Exactas y Naturales, Pontificia Universidad Católica del Ecuador, Quito, Ecuador,; 3 Department of Microbiology and Immunology, The University of Melbourne, Bio21 Institute and Peter Doherty Institute, Melbourne, VIC, Australia,; 4 Department of Applied Mathematics, University of Colorado Boulder, Boulder, CO, United States,; 5 Department of Computer Science, University of Colorado Boulder, Boulder, CO, United States,; 6 BioFrontiers Institute, University of Colorado Boulder, Boulder, CO, United States

**Keywords:** malaria elimination, antigenic diversity, disease transmission, outbreak, genomic epidemiology, molecular surveillance, *var* DBLα, *Plasmodium falciparium*

## Abstract

To better understand the factors underlying the continued incidence of clinical episodes of falciparum malaria in E-2025 countries targeting elimination, we characterized the molecular epidemiology of *Plasmodium falciparum* disease transmission after a clonal outbreak in Ecuador. Here we study disease transmission by documenting the diversity and population structure of the major variant surface antigen of the blood stages of *P. falciparum* encoded by the *var* multigene family. We used a high-resolution genotyping method, “*var*coding”, involving targeted amplicon sequencing to fingerprint the DBLα encoding region of *var* genes to describe both antigenic *var* diversity and *var* repertoire similarity or relatedness in parasite isolates from clinical cases. We identified nine genetic *var*codes in 58 *P. falciparum* isolates causing clinical disease in 2013–2015. Network analyses revealed that four of the *var*codes were highly related to the outbreak *var*code, with identification of possible diversification of the outbreak parasites by recombination as seen in three of those *var*codes. The majority of clinical cases in Ecuador were associated with parasites with highly related or recombinant *var*codes to the outbreak clone and due to local transmission rather than recent importation of parasites from other endemic countries. Sharing of types in Ecuadorian *var*codes to those sampled in South American *var*codes reflects historical parasite importation of some *var*codes, especially from Colombia and Peru. Our findings highlight the translational application of *var*coding for outbreak surveillance in epidemic/unstable malaria transmission, such as in E-2025 countries, and point to the need for surveillance of local reservoirs of infection in Ecuador to achieve the malaria elimination goal by 2025.

## Introduction

A recent global push for national malaria elimination in 21 countries by 2020 led to three being declared malaria-free (Algeria, El Salvador and Paraguay), but the target was not met for the remaining countries (1, 2). These countries are now part of a renewed initiative launched by the WHO in 2021 supporting a total of 25 countries, known as E-2025 countries, with the shared goal to eliminate local transmission of malaria by 2025 by reducing the incidence of indigenous or locally transmitted cases to zero (2). These countries include Mexico, Panama, Ecuador, South Africa, Eswatini, Thailand, Malaysia, among others. Malaria transmission in many of these countries is epidemic/unstable with risks of outbreaks, parasite importation, and resurgent malaria (3–5).

Ecuador, one of the nine E-2025 countries in Latin America, did not meet the 2020 elimination target due to malaria resurgence since 2015 (2, 6, 7). Malaria elimination efforts in Ecuador have largely focused on tropical areas, specifically the northwest coast and the Amazon region (8–12). These areas border non E-2025 countries, Colombia and Peru, that still have endemic and moderate to low transmission (13–20). Clinical cases caused by *P. falciparum* are mostly concentrated in the northwest coast and Ecuador has epidemic/unstable *P. falciparum* transmission, with *P. vivax* being the dominant species. Determining whether the continued incidence of *P. falciparum* clinical cases is due to imported or locally acquired parasites is of key public health interest to better understand disease transmission patterns and aid decision-making to allocate limited resources to achieve the E-2025 elimination goal.

Application of genotyping methods to epidemiological analyses can aid public health responses in such settings by informing on parasite diversity and relatedness to identify imported cases and characterize residual and/or resurgent disease transmission patterns with higher resolution than routine case incidence monitoring (21, 22). Such epidemiological surveys can be impactful in delineating malaria transmission hotspots, if any, within an endemic area. Molecular surveillance to characterize malaria transmission patterns has relied on genotyping neutral molecular markers such as microsatellites or single-nucleotide polymorphisms (SNPs) as more cost-effective approaches than whole genome sequencing. Of relevance to low or epidemic transmission, neutral variation has been commonly used to inform about clonality and relatedness as well as track genomes spatially. Genes under selection can provide an alternative but complementary view of microevolution (23–25).

**FIGURE 1:**
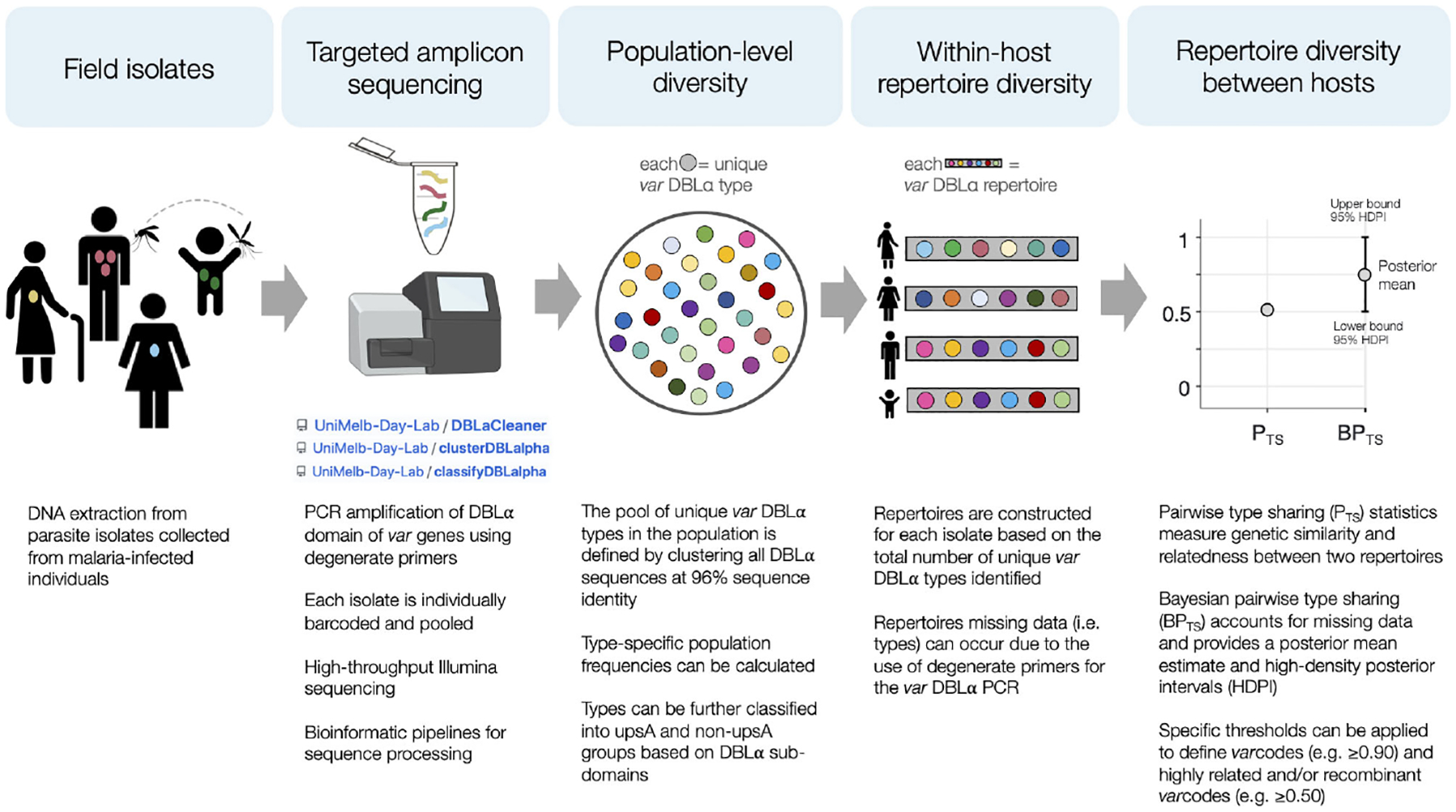
Schematic diagram of the *var*coding approach. For more details about each step, see Methods. The illumina sequencer stock image was created with BioRender.com.

Molecular surveillance based on diversity of the genes encoding the major surface antigen of pathogens is a well-established microbiological paradigm typically used in virology (e.g. Influenza A, HIV-1, SARS-CoV-2) and bacteriology (e.g. *Neisseria* spp, *Streptococcus pyogenes*) because genotyping such antigens provides important information on recombination rates, the factors associated with these recombination events, and transmission dynamics. Employing this surveillance approach is however more complex in falciparum malaria where the major variant surface antigen of the blood stages known as PfEMP1 is encoded by the *var* multigene family with 40–60 *var* genes per genome (26) and extensive diversity of *var* genes exists in parasite populations (27). Indeed, *var* diversity has been shown to be correlated with transmission intensity with the highest diversity, in the order of tens of thousands of variants, seen in African populations and the lowest in the Americas, in the order of hundreds (27–31). *Var* genes and repertoires diversify by meiotic recombination during the obligatory sexual phase of the life cycle in the mosquito as well as by mitotic recombination. We define a fingerprint of the *var* repertoire of an isolate by genotyping the highly diverse DBLα tag of the *P. falciparum var* multigene family (29–33). This fingerprinting method we call “*var*coding” requires a single PCR with degenerate primers followed by amplicon deep sequencing. We sample DBLα diversity of *P. falciparum* within and between human hosts, as well as measure similarity or relatedness between *var* repertoires we call *var*codes. Bayesian statistics are used to account for variable sampling of all members of the multigene family (genomes contain 40–60 *var* genes) in field samples when amplifying low-quality parasite DNA with degenerate primers and to quantify the uncertainty around relatedness estimates (Figure 1). This analytical approach was designed specifically for *var* genes under immune selection where alleles per se cannot be assigned since chromosomal positions are unknown, and commonly used approaches to infer relatedness such as identity-by-descent (IBD) are not straightforward to apply due to the complexities of this multi-copy *var* gene family.

Here, we apply *var*coding for the first time in unstable, epidemic malaria to look at diversity and population structure of these immune evasion genes. Specifically, we characterize the transmission of clinical *P. falciparum* cases in Ecuador during and up to two years after an outbreak in 2012–2013, which was previously found to be clonal by microsatellite genotyping (i.e., caused by a single parasite lineage) (19). Analysis of *var*code relatedness networks allowed us to define different genomic parasite lineages (or *var*codes) circulating locally, as well as detect signatures of highly related parasites and possibly recently recombined genomes with respect to *var* repertoires. Parasites with the *var*code of the outbreak clone or recombinant/highly related *var*codes relative to the outbreak clonal lineage were predominantly associated with the continued incidence of clinical cases after the outbreak. Hot spots for *var*code diversity were identified suggestive of the existence of local reservoirs of infection. Further comparative analyses to published data from historical South American isolates (30) elucidated possible origins of Ecuadorian parasites, demonstrating that the majority of clinical cases were due to local transmission and not recent importation.

## Methods

### Study design and sample collection

In this molecular epidemiological study, we examined *P. falciparum* isolates collected from 2013–2015 in Ecuador from individuals of all ages presenting with uncomplicated malaria cases confirmed by microscopy and/or PET-PCR (34). Details on the study population and data collection have been previously published (19, 35, 36). Briefly, these samples were collected during passive surveillance by the Ecuadorian Ministry of Health from consenting participants who lived in the areas where the samples were taken, were over 2 years old, agreed to participate and provided a blood sample (either venous blood or dried blood spot) and answered a basic demographic questionnaire (including places recently travelled and their address). Genomic DNA was extracted from venous blood or dried blood spots using a QIAamp DNA MINI KIT (QIAGEN, USA) as recommended by the manufacturer. The study was approved by the ethics committees at the Pontificia Universidad Católica del Ecuador (Quito, Ecuador) and The University of Melbourne (Melbourne, Australia).

### *Var* DBLα amplification and sequencing

*Var* genotyping PCR, sequencing details, and related data processing steps have been previously published (33, 37). Briefly, for each *P. falciparum* isolate, a single-step PCR to amplify the DBLα domains of *var* genes was performed using universal degenerate primers targeting homology blocks D (forward primer) and H (reverse primer) originally described in (38), with the addition of 10bp GS FLX Titanium multiplex identifier primer sequence for barcoding (39). The PCR reaction was prepared in a total volume of 40μl, containing 3μL of genomic DNA, MgCl_2_ at a final concentration of 2mM, dNTPs at a final concentration of 0.07mM, each primer at a final concentration of 0.375mM and 3 units of Flexi DNA Taq polymerase (Promega). The cycling conditions were as follows: initial denaturation step of 2 min at 95°C was followed by 30 cycles of: annealing for 40 seconds at 95°C, extension for 90 seconds at 49°C, denaturation for 90 seconds at 65° C, and then a final extension step of 10 min at 65°C. The resulting individually barcoded DBLα amplicons (approximately 450–700bp in length) were pooled equimolarly and barcoded libraries were prepared using the KAPA Low-Throughput Library Preparation Kit (Kapa Biosystems). The libraries were sequenced on a MiSeq Illumina platform using the 2×300bp paired-end protocol and MiSeq Reagent kit v3 chemistry (Australian Genome Research Facility, Melbourne, Australia).

### *Var* DBLα data processing

The raw illumina sequence data was then cleaned and processed using the DBLaCleaner pipeline [(37, 40), http://github.com/Unimelb-Day-Lab/DBLaCleaner]. Our customized bioinformatic pipeline has been described in detail in (37, 40). Briefly, we de-multiplexed and merged the paired reads as well as removed low-quality reads and chimeras using several filtering parameters (see Supplementary Figure S1 for details). This pipeline resulted in 2,141 cleaned DBLα sequences (Supplementary Figure S1). To identify distinct or unique DBLα types (i.e., unique genetic variants), we clustered the DBLα sequences from Ecuadorian *P. falciparum* isolates with 5,699 previously published (30) DBLα sequences from other South American countries (Colombia (N=21 isolates), French Guiana (N=76 isolates), Peru (N=21 isolates), and Venezuela (N=10 isolates) at the standard 96% sequence identity (32) using the clusterDBLalpha pipeline (http://github.com/Unimelb-Day-Lab/clusterDBLalpha). We further curated our dataset by translating the DBLα types into amino acid sequences using the classifyDBLalpha pipeline (http://github.com/Unimelb-Day-Lab/classifyDBLalpha) and removing any DBLα types that were non-translatable (N=4). All the cleaned DBLα sequences in this study (Supplementary Figure S1) have been submitted to the DDBJ/ENA/GenBank (BioProject Number: PRJNA642683). A tutorial on the DBLα processing pipelines can be found at http://github.com/Unimelb-Day-Lab/tutorialDBLalpha.

In addition, any *P. falciparum* isolate with low sequencing quality (< 10 DBLα types) was removed from the analysis. Therefore, from a total of 70 genotyped *P. falciparum* Ecuadorian isolates, 12 P*. falciparum* isolates with low DNA quality and/or poor sequencing quality were removed and we obtained *var* DBLα data for 58 isolates (82.9%). A total of 543 unique DBLα types identified in the 186 South American *P. falciparum* isolates [N = 58 Ecuadorian *P. falciparum* isolates from this study, N = 128 South American *P. falciparum* isolates previously published in (30)] were used for subsequent *var* analyses at the regional-level, and only the 195 unique DBLα types identified in Ecuador were used for Ecuador-specific analyses. To evaluate how well we sampled the true pool of *var* DBLα diversity (i.e., the true number of genetic variants circulating) in Ecuador and in South America, we used the R package *vegan* (41) to generate species accumulation curves where the number of unique DBLα types are plotted as a function of the number of sampled sequences. Plateauing of this curve indicates saturation and robust sampling of the diversity pool (i.e. sampling more sequences will not identify any new types).

### Genetic relatedness analyses

#### Measuring pairwise type sharing

To estimate *var*code similarity or relatedness between all isolate pairs, we calculated the similarity index Pairwise Type Sharing (P_TS_) (28), as adapted by He et al. (37) to account for differences in DBLα sampling across isolates (i.e, differences in isolate repertoire sizes), and unbiased Bayesian pairwise type sharing estimates (BP_TS_) to further account for uncertainty in P_TS_ estimates. P_TS_ represents the proportion of shared DBLα types between an isolate pair. It is calculated directionally by dividing the number of shared DBLα types, *N*_*s*_, between two isolates *a* and *b*, by the total number of DBLα types in each isolate or repertoire size, *N*_*a*_ or *N*_*b*_. Thus, for each pair of isolates we calculate *P*_*TS(a,b)*_ *= N*_*s*_*/N*_*a*_ and *P*_*TS(b,a)*_ *= N*_*s*_*/N*_*b*_.

*Var*code relatedness can also be explored more rigorously with unbiased Bayesian pairwise type sharing (BP_TS_) (42, 43). This approach uses Bayesian inference methods, which estimate repertoire overlap and uncertainty, and uses them in a subsequent P_TS_ calculation, carrying that uncertainty forward. The prior distribution for repertoire size, used in inference, was informed by observations as follows. First, the median observed repertoire size in Ecuadorian isolates was 37 types, ranging from 11 to 43 (Supplementary Table S1). Second, the number of expected *var* genes with DBLα domains from whole genome sequencing data of the Honduran laboratory reference strain HB3 was 42 (26) and 50 *var* genes based on long-read PacBio sequencing of HB3 (44). And third, based on our sequencing data of 37 technical replicates of HB3, the median repertoire size or number of DBLα types per isolate was 39 (range 36–41 types), with 40 types consistently identified in the majority of replicates (range 21–37 replicates). We therefore used a uniform prior on repertoire sizes between 40 and 50 types, combined with the general Bayesian repertoire overlap framework (42) to produce unbiased estimates (posterior means). These were used to confirm our P_TS_ estimates. As expected, the P_TS_ and BP_TS_ estimates were positively correlated (Pearson’s correlation coefficient = 0.919, *p* < 0.001, Supplementary Figure S3). To measure uncertainty in central estimates, we computed a 95% highest density posterior interval (HDPI), a Bayesian version of confidence intervals, for each pairwise estimate. Like a frequentist confidence interval, the width of the HDPI provides a measure of uncertainty of each pairwise comparison. All posteriors were sampled using Markov chain Monte Carlo.

### Interpretation of varcode relatedness measures

Every parasite isolate was compared to every other parasite isolate in the population to determine the proportion of shared DBLα types. Theoretically, pairwise comparisons resulting in P_TS_ of 0 (0% shared types), 0.5 (50% shared types), and 1 (100% shared types) will reflect genetically distinct isolates with different *var* repertoires, isolates with recombinant or highly related *var* repertoires, and clones or genetically identical isolates with the same *var* repertoire, respectively. In practice, however, in low-transmission areas often “fixed” relatedness thresholds may obscure true relatedness estimates because of inbreeding events and thus require confirmation by measuring the uncertainty around each estimate with methods like our Bayesian inference of P_TS_. We applied this approach to define “*var*codes” as groups of isolates sharing ≥90% of their DBLα types (P_TS_ ≥ 0.90), identifying putatively identical genomes within the margin of error of detection of 1–5 DBLα types in an isolate. We confirmed our interpretations of *var*codes, recombinants/highly related, and genetically distinct isolates at the thresholds of 0, 0.5 and 0.90–1, respectively, by comparing to unbiased BP_TS_ estimates (posterior means) and examining the HDPI.

### Visualization of varcode relatedness networks

To visualize the *var*code relatedness between isolates as determined by P_TS_ or BP_TS_, we constructed networks using the R packages *ggraph* (45) and *tidygraph* (46) where isolates are depicted as nodes and edges as the P_TS_ or BP_TS_ values at a given threshold. The R package *ggspatial* (47) was used to plot spatial networks using latitude/longitude coordinates for sampling location. To visualize *var*code relatedness of parasites over time we used the R package *gganimate* (48) to construct spatiotemporal relatedness networks. We generated a clustered heatmap based on the presence/absence matrix of DBLα types to visualize the genetic profiles of each isolate in Ecuador and each country in South America using the R package *pheatmap* (49) and the “complete” clustering method. Unrooted neighbor-joining phylogenetic trees based on pairwise genetic distance (calculated as 1-P_TS_) were constructed using the R packages *ape* (50) and *ggtree* (51, 52).

### Statistical analysis

We used R version 3.5.2 (53), base R, and the R packages *dplyr* (54), *epiR* (55) for all analyses. We used chi-squared tests for univariate analyses of categorical variables to compare proportions and for non-parametric tests to compare distributions of continuous variables between two groups (Mann-Whitney U test) or among *k* groups (Kruskal-Wallis test), with a Bonferroni correction for multiple comparisons.

## Results

### Limited *var* diversity in Ecuadorian *P. falciparum* populations

**FIGURE 2:**
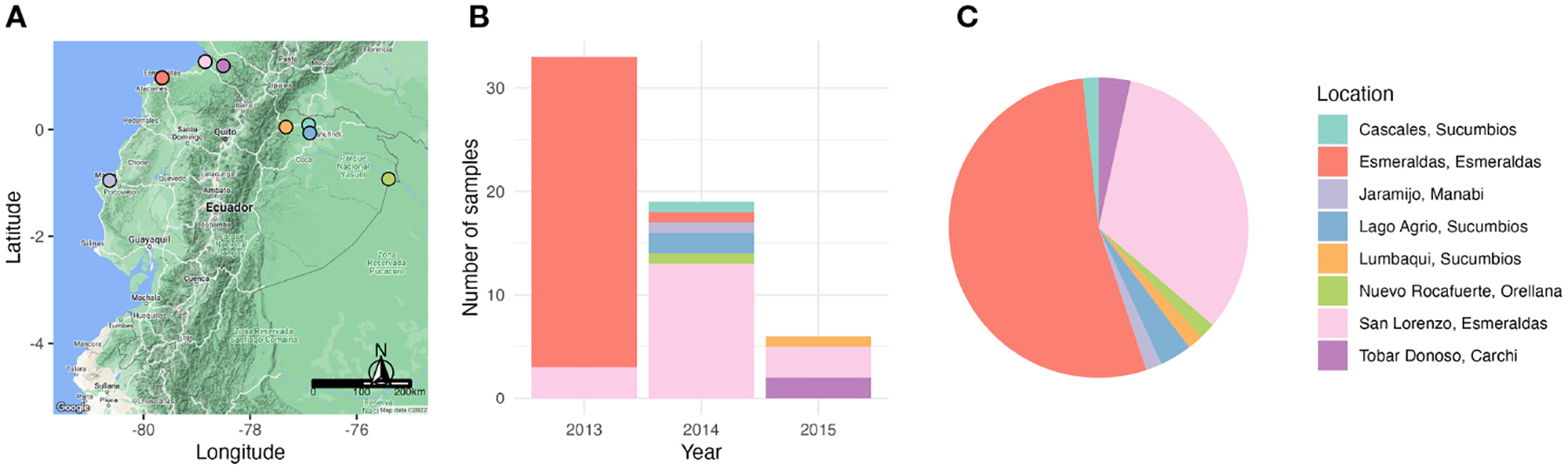
Sampling of *P. falciparum* isolates across endemic areas of Ecuador and over a period of three years (2013 – 2015). **(A)** Map of Ecuador depicting the sampling locations during the study. **(B)** Bar plot showing the number of *P. falciparum* positive samples collected in each year and their respective sampling locations. **(C)** A pie chart showing the proportion of *P. falciparum* positive samples in each sampling location. Every location is indicated with a different color.

Diversity of *var* genes was assessed by *var*coding for 58 P*. falciparum* isolates that were collected between 2013 and 2015 from individuals of all ages experiencing clinical malaria (Figure 2). These isolates represent 21% of the total cases reported in 2013, 61% in 2014, and 3% in 2015 (60% of the cases reported in January, 7% in May and 8% in November 2015). Overall, we identified 195 unique DBLα variants or types from 2,141 DBLα sequences (Supplementary Figure S1) from the 58 isolates, representative of the diversity circulating in Ecuadorian *P. falciparum* populations, as indicated by sampling accumulation curves approaching a plateau (Supplementary Figure S2).

### Defining nine distinct *var*codes circulating in Ecuador and describing spatiotemporal disease transmission trends

**FIGURE 3:**
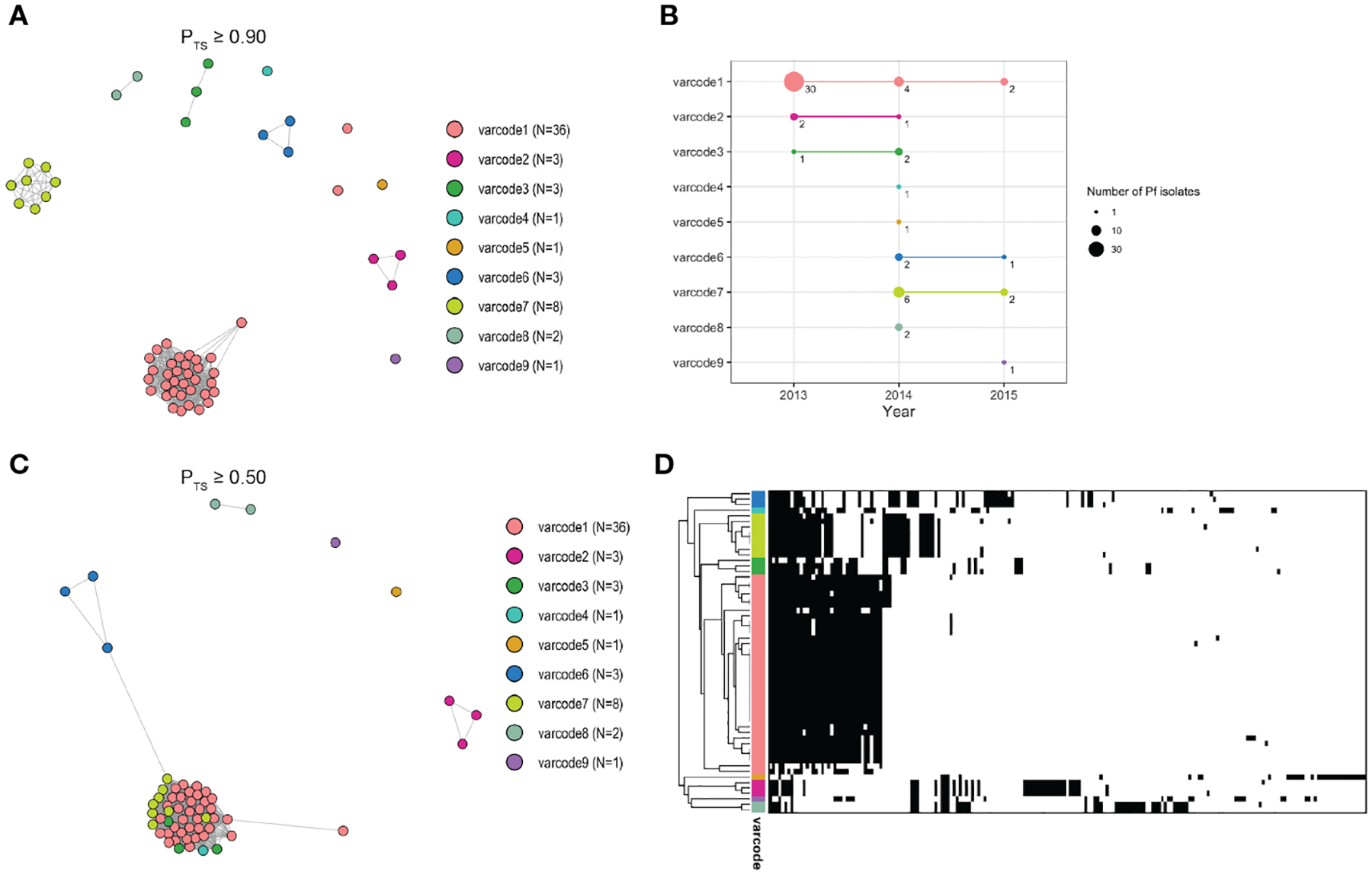
*Var*code relatedness networks in Ecuador **(A)** A network visualization of the *var*code relatedness of *P. falciparum* isolates at the threshold of P_TS_ ≥0.90 to define *var*codes (see Methods). Every node represents a *P. falciparum* isolate and an edge represents the P_TS_ value between two particular nodes/isolates. Isolates that cluster together (i.e., connected by edges) are considered to have the same *var*code. Each color represents a different *var*code. Two *P. falciparum* isolates belonging to outbreak *var*code1 appear as outliers in the network due to undersampling of their DBLα types. N refers to the number of isolates. For comparison to BP_TS_ estimates see Supplementary Figures S4, S5. **(B)** The number of *P. falciparum* isolates within each *var*code (i.e., size of circle) in each year and their persistence over time. The three *var*codes (*var*code1, *var*code2, *var*code3) identified in 2013 were identified again in 2014, and outbreak *var*code1 was identified again in 2015. Two *var*codes identified in 2014 (*var*code6, *var*code7) were also identified in 2015. **(C)** A network visualization of the genetic relatedness of *var*codes at the threshold of P_TS_ ≥0.50 to discriminate between highly related/recombinant *var*codes and genetically distinct *var*codes. Isolates/*var*codes that cluster together (i.e., connected by edges) represent highly related/recombinants. For comparison to BP_TS_ estimates see Supplementary Figures S5, S6. **(D)** A clustered heatmap showing the genetic profiles of each *P. falciparum* isolate with rows representing each isolate and columns representing each DBLα type. Black and white denote the presence and absence of each type, respectively. Isolates that clustered together were more genetically similar (i.e., the same DBLα types were present). Similarly, *var*codes that clustered together were more genetically similar (e.g., highly related/recombinants).

**FIGURE 4:**
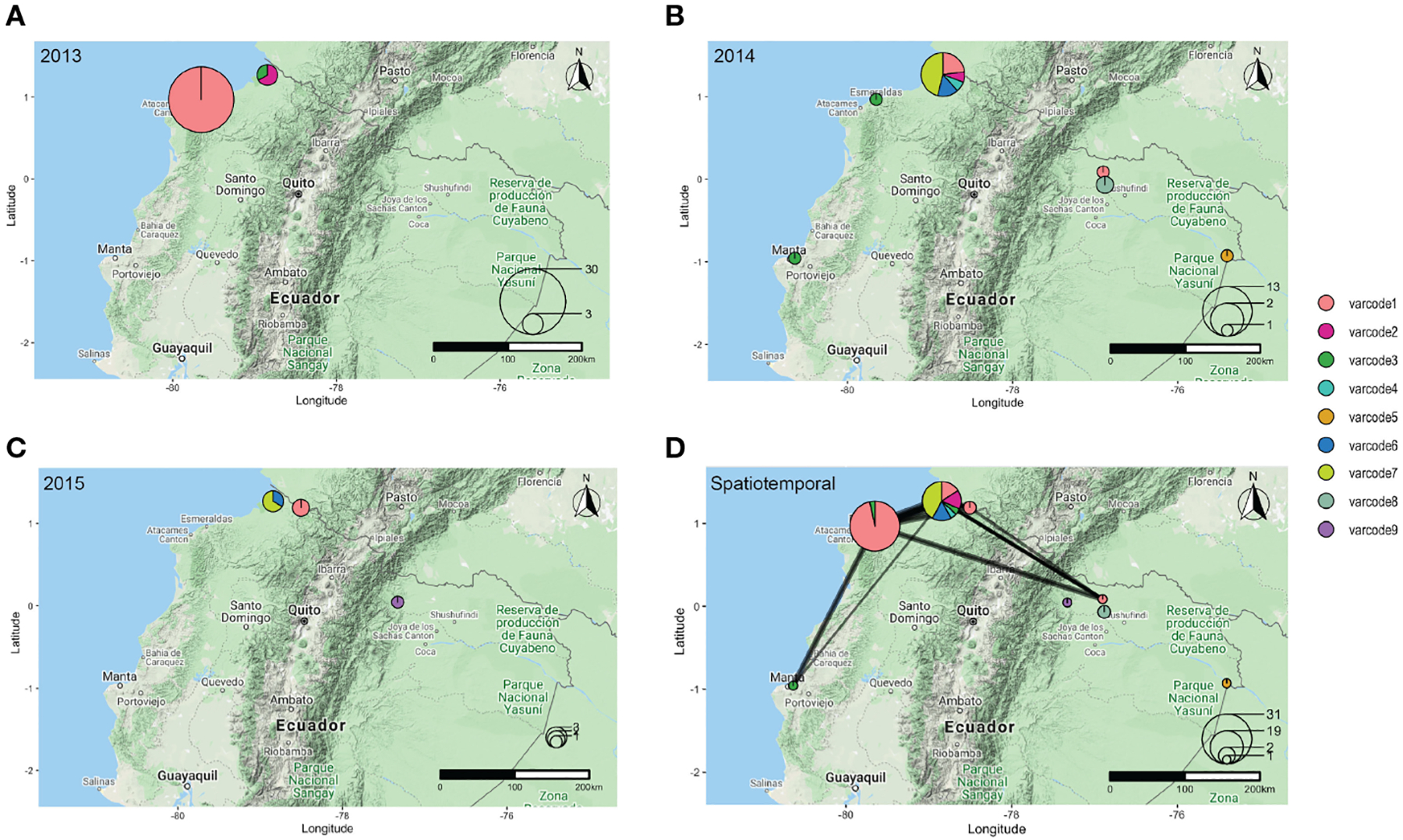
The spatial distribution of *var*codes in **(A)** 2013, **(B)** 2014, **(C)** 2015, with the size of the circle representing the number of *P. falciparum* isolates sampled in a given location in each year and the pie chart depicting the proportion of each *var*code identified. **(D)** Spatiotemporal *var*code relatedness network between 2013–2015. Every node represents a sampling location, the size of the circle represents the number of *P. falciparum* isolates sampled in each location, the pie chart depicts the proportion of each *var*code identified, and the weighted edges show genetically related *var*codes (P_TS_ ≥0.50).

To define distinct *var*codes circulating locally in Ecuador, we estimated relatedness between two isolates’ *var*codes by calculating the similarity index, Pairwise Type Sharing (P_TS_) and constructed relatedness networks to identify genomes with the same *var*codes (P_TS_ ≥0.90). We confirmed this by comparing *var*codes derived from P_TS_ to those derived from posterior mean BP_TS_ estimates (Supplementary Figure S3), which include statistical uncertainty, and by examining the lower and upper bounds of the 95% highest density posterior intervals (HDPIs). This revealed nine genetically distinct *var*codes in this study with 36 isolates having *var*code1 (representing the clonal outbreak in Esmeraldas City, salmon pink *var*code Figures 3 and Figure 4A) and at the other extreme *var*code4, *var*code5, *var*code9 were seen only once (Figure 4A, S4A). Our definition of *var*codes proved to predict genomic lineages (identity-by-descent ≥0.99) based on published whole genome sequence data in the case of 30 of the isolates (six *var*codes) that were analyzed by both WGS (56) and *var*coding. As expected, the outbreak *var*code1 was clonal (indicated by HDPIs that included 1; Figure S5B), as previously demonstrated by microsatellite genotyping (19) and more recently by WGS (56). The outbreak *var*code1 identified in Esmeraldas City was also identified in San Lorenzo, Esmeraldas (~150km from Esmeraldas City) in 2013, then Cascales, Sucumbios (>300km away) in 2014, and then in Tobar Donoso, Carchi (~150km away) in 2015 (Figure 4B). Overall, persistent disease transmission of the same *var*codes was observed both over time (Figure 4B) and large distances (Figures 3A–C). The median time between first and last identification of the same *var*codes with any clinical case was 216 days or approximately 7 months (range = 190 – 823 days) during the study period. This is in line with reports in Peru (57) and Colombia (18, 58) where clonal parasites were shown to be circulating up to five and eight years after first identification, respectively.

### *Var*code relatedness networks reveal signatures of highly related parasites and local disease transmission

We next constructed spatiotemporal *var*code relatedness networks to explore signatures of local disease transmission after the outbreak. Figure 4C shows that 15 isolates with four *var*codes (*var*code3, *var*code4, *var*code6, *var*code7) clustered with the outbreak *var*code1 at the threshold of P_TS_≥0.50 (Figure 4C). The remaining 7 isolates with different *var*codes did not cluster in the network and were genetically distinct. These patterns were confirmed using BP_TS_ estimates based on posterior means (Supplementary Figure S6A) and their corresponding 95% HDPIs (Supplementary Figures S5C–F, S6B, C).

To better understand the *var*code relatedness signatures in the “outbreak cluster” we examined both P_TS_ and BP_TS_ estimates. In some instances, highly related *var*codes shared ~50% of the outbreak DBLα types, such as the outbreak *var*code1 with *var*codes3,4,6,7 (median P_TS_ range=37–58%, median BP_TS_=45–70%, Figures S5C, D). This level of *var*code relatedness would be consistent with the generation of a new *var*code through outcrossing by conventional meiosis resulting in recombinant *var* repertoires of the outbreak clone. When examining the pairwise combinations of *var*codes 3,4,6,7, sharing of types also indicates high relatedness (median P_TS_ range=27–50%, median BP_TS_=32–64%, Figures S5C, D), pointing to the possibility of a prior cross followed by a backcross given the overall limited *var* diversity in the parasite population. However, the timing of such crosses is unknown. Interestingly, all the possibly recombinant and highly related *var*codes (*var*codes3,4,6,7) were identified in San Lorenzo with *var*code7 being the main one, indicating this area is a transmission hot spot and a reservoir of parasites with diverse *var* repertoires (Figure 3D).

To further confirm the observed *var*code relatedness signatures, we constructed a clustered heatmap to visualize the genetic profiles of each isolate based on the presence/absence of the 195 DBLα types, such that genetically similar isolates cluster together (Figure 4D). This analysis confirmed that in the case of isolates identified as highly related to the outbreak *var*code1, a proportion of outbreak DBLα types as well as different DBLα types were present. By contrast, the genetic profiles of isolates with *var*codes 2, 5, 8, and 9 had more different DBLα types to all other *var*codes, i.e., parasites with genetically distinct *var*codes, with sharing of only <30% of types in most instances (median P_TS_ range= 2–29% except for 2 pairwise comparisons 34–37%; median BP_TS_ range=4–30% except for 5 pairwise comparisons 31–41%, Figures S5E, F).

### Parasites with highly related/recombinant *var*codes were most frequently associated with *P. falciparum* clinical episodes following the 2012–13 outbreak

Prior to the outbreak there had been a steady decline in clinical cases; however, increased incidence of disease occurred after the outbreak. Therefore, we analyzed trends in the epidemiology of *P. falciparum* cases that occurred post-outbreak to understand if there were any key risk factors. Of the 25 individuals in our study that had a clinical *P. falciparum* episode post-outbreak, we had age data for 18 individuals (72%, Table 1). There was no significant difference (Kruskal-Wallis test, *p* = 0.65) in the median age of individuals experiencing clinical episodes caused by parasites with the outbreak *var*code1 (19 years, range = 19 – 57 years, N = 3 patients), a highly-related/recombinant of *var*code1 (i.e., *var*codes 3, 4, 6, or 7) (25 years, range = 17 – 58 years, N = 13 patients), or by a different *var*code (34.5 years, range = 32 – 37 years, N = 2 patients). A greater diversity of *var*codes (1–9 inclusive) was associated with incidence of clinical malaria post-outbreak than during the 2013 outbreak (*var*codes 1,2,3). Indeed in 2014, 79% of the cases sampled were caused by either parasites with the outbreak *var*code1 (21%) or with any of the four highly-related/recombinants of *var*code1 (58%). The trend was similar in 2015 with 83% of the cases sampled caused by either parasites with the outbreak *var*code1 (33%) or with any of the four highly-related/recombinants of *var*code1 (50%), although our sampling of reported cases in 2015 after January was limited. Importantly, overall, we found that 80% of the cases sampled after the outbreak were caused by either parasites with the outbreak *var*code1 (24%) or with any of the four highly-related/recombinants of *var*code1 (56%), especially with *var*code7 (representing 57% of the cases caused by parasites with highly-related/recombinant *var*codes). Thus, disease transmission after the outbreak in 2014 and into 2015 was predominantly associated with parasites with highly related/recombinant *var* repertoires of the outbreak clone, with most of these cases occurring in the San Lorenzo hotspot.

### Comparative analyses to South America: elucidating possible origins of Ecuadorian *var*codes and signatures of historical importation

**FIGURE 5:**
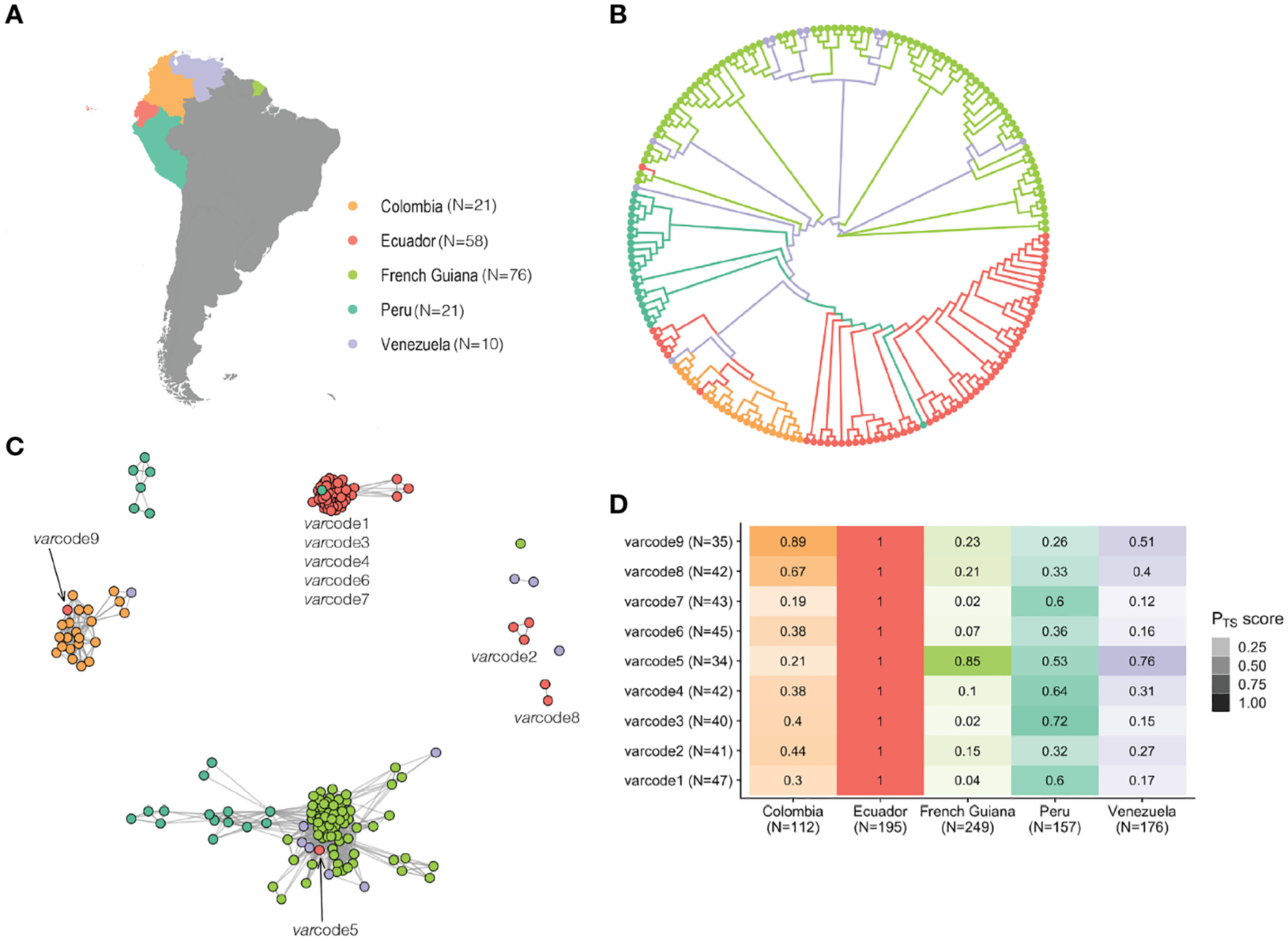
South American *var*code relatedness networks provide insights into the origins of the Ecuadorian *var*codes and reveal signatures of historical importation. **(A)** A map showing the study sites across South America. The dates of sample collection in these countries occurred from 2002 to 2008, around five to thirteen years prior to the sample collection of the Ecuadorian isolates. N refers to the number of isolates. **(B)** An unrooted neighbor-joining tree shows the relatedness patterns among South American *P. falciparum* isolates neighbor-joining tree shows the relatedness patterns among South American *P. falciparum* isolates based on genetic distance (1-P_TS_, see Methods). **(C)** A network visualization of the *var*code relatedness of Ecuadorian and South American isolates at the threshold of P_TS_ ≥0.50. Every node represents a *P. falciparum* isolate and an edge represents the P_TS_ value between two particular nodes/isolates. Isolates/*var*codes that cluster together (i.e., connected by edges) are genetically related. **(D)** Relatedness of Ecuadorian *var*codes to *P. falciparum* populations from South America. The relatedness of each *var*code was measured by first concatenating all possible *var* DBLα types that were identified in the *P. falciparum* isolates comprising each *var*code as well as concatenating all *var* DBLα types that were identified in each country. Then P_TS_ was calculated between each *var*code and each country. The color gradient denotes the P_TS_ value for a particular comparison, with darker shades showing higher relatedness and the different colors corresponding to the different country comparisons. N refers to the number of *var* DBLα types.

We next examined the possible origins of the *var*codes circulating locally in Ecuador by comparing to the only published *var* DBLα dataset from the region (30), comprising 128 *P. falciparum* isolates collected from South American *P. falciparum* populations in 2002–2008 from countries with higher malaria transmission compared to Ecuador (Figure 5A). In all countries (except some isolates in French Guiana), the number of DBLα types per isolate repertoire, a proxy for complexity of infection (COI, i.e. the number of *P. falciparum* genomes in an infection), was indicative of only one *P. falciparum* genome infecting an individual (i.e., repertoire size ≤ 50 or COI=1, Supplementary Figure S9; Supplementary Table S1). Despite the difference in sampling years, the 543 unique DBLα types identified in these 186 isolates represent the diversity circulating in South American *P. falciparum* populations, as indicated by DBLα sampling accumulation curves approaching saturation (Supplementary Figure S7). An unrooted phylogenetic neighbor-joining tree showing pairwise genetic distance (1-P_TS_) revealed distinct, country-specific clusters of isolates with related *var*codes (Figure 5B), consistent with previous analyses demonstrating geographic population structure (30). Thus, despite the relatively low *var* diversity, there is sufficient resolution to differentiate between the parasite populations from different South American countries and to explore the possible geographic origins at the level of country-specific DBLα types but also the unique combinations of these types as *var*codes.

*Var*coding resolved a total of 97 *var*codes in South America (P_TS_ ≥ 0.90), ranging from nine in Ecuador and Venezuela, to 56 *var*codes in French Guiana and the *var*codes were country-specific since none of the isolates from different countries clustered with isolates from another country at this threshold (Supplementary Figure S8). However, although identical *var*codes were not identified in multiple countries, we were interested in examining *var*code relatedness. To assess the overall sharing of types in the *var*codes identified in Ecuador to those sampled in other South American parasite populations, we aggregated all possible DBLα types identified in the isolates comprising each Ecuadorian *var*code (range: 34–47 types per varcode) and all DBLα types seen in any isolate from a given country (range: 112–249 types per country). The overall sharing of types was high, with the highest median sharing with Peru (53%, range: 26–72%) followed by Colombia (39%, range: 19–89%) and Venezuela (27%, range: 12–76%), and only 10% with French Guiana (range: 3–86%) except for *var*code5 (86%) (Figure 5D).

Next we estimated P_TS_ between all possible isolate pairs to examine whether any of the Ecuadorian parasites had *var*codes that were genetically related to these historical South American parasites (P_TS_ ≥ 0.50) and constructed regional *var*code relatedness networks (Figure 5C). We identified a genetically related Peruvian *P. falciparum* isolate that clustered with the “outbreak cluster”, but especially with outbreak *var*code1 (P_TS_ = 0.66–0.75 with *var*code1 isolates). This is consistent with previous analyses using microsatellites showing the outbreak source was possibly a residual parasite lineage circulating in Peru in 1999–2000 and in Ecuador in the early 1990s (13, 19). More recent whole genome sequence data also points to the outbreak *var*code1 circulating in Colombia in the early 2000s (56). No other South American isolate clustered with the “outbreak cluster” based on our network analysis, suggesting that there may be other locally circulating parasites in unidentified reservoirs in Ecuador, e.g. the parental *var*codes of the possible recombinants. In the case of *var*code3, the WGS study showed that isolates with *var*code1 and *var*code3 belong to distinct genomic lineages but are identical by descent across 80% of the genome thus belong to a “super-cluster” (56), which is consistent with our BP_TS_ estimates of 70–84% type sharing (Figure S5D). Additionally, the WGS study identified *var*code6 and *var*code7 in Colombian isolates from 2014–2016 and in 2016, respectively, but *var*code4 was not identified in any of their Colombian isolates (56).

We were also interested to better understand the origins of those *var*codes that did not cluster with the “outbreak cluster” since historically they may have been imported and may represent other locally circulating parasites that were not a direct result of onward transmission after the outbreak. We found evidence of two putative importations of parasites from neighboring countries, *var*code9 from Colombia (Case EC53, Table 1), based on both genetic and epidemiologic data, and *var*code5 (Case EC40, Table 1) the origin of which was less clear. The epidemiological data for the putative infection location was recorded as Peru (Case EC40, Table 1), however based on our data this *var*code is more related to historical *P. falciparum* populations from French Guiana and Venezuela than Peru. The remaining Ecuadorian isolates with *var*codes 2 and 8 did not cluster with any other South American isolate. The epidemiological data for the putative infection location for *var*code8 was recorded as Colombia (EC49 and EC50, Table 1), but these isolates did not cluster with Colombian isolates in our network analysis. Sharing of DBLα types in these *var*codes with Colombian types was high (44% for *var*code2 and 67% for *var*code8, Figure 5D), providing evidence that parasites with *var*code2 and *var*code8 may represent residual parasites historically imported from Colombia. This is consistent with WGS data for isolates with *var*code2, demonstrating they are identical by descent (IBD>0.99) with Colombian isolates from the early 2000s (56), suggesting past rather than recent importation. It is worth noting that *var*code2 was identified in the hotspot of San Lorenzo, Esmeraldas confirming that this location may act as a reservoir of parasites with diverse *var* repertoires, as described above.

## Discussion

Our investigation of the spatiotemporal incidence of clinical episodes of *P. falciparum* in Ecuador by *var*coding supports the view that disease transmission after the 2012–2013 outbreak was sustained by parasites circulating in Ecuador, some of which may have had historical origins in neighboring countries. We observed persistence of the outbreak clonal lineage (identified with the same *var*codes) and parasites with highly related *var*codes predominantly associated with clinical disease after the outbreak. The observed *var*code relatedness signatures may point to potential outcrossing of the outbreak lineage with other locally circulating parasites. Whether these recombinant *var*codes resulted from sexual recombination events between parasites with outbreak *var*code1 and genetically distinct parasites that were already circulating at low levels and/or in asymptomatic reservoirs in Ecuador [e.g (12)] or those that were previously imported, could not be ascertained from the current study population. Our results point to the need for resources to be focused locally in Ecuador to uncover the circulating reservoirs of infection. In this context, *var* genes will undoubtedly play a role in the persistence and virulence of these parasites. A role for human mobility must also be considered in the spread of *P. falciparum* in Ecuador, as parasites with the same *var*codes were also observed across large distances (~150–300km) and two putative importations from Colombia and Peru were identified based on both epidemiological and *var*coding data.

San Lorenzo was found to be a transmission hotspot likely due to mining activities and occupation-related travel in these areas, as well as its proximity to the Ecuador-Colombia border (4, 35, 59–61). These findings point to San Lorenzo for both genetic surveillance and targeted interventions. Importantly, our results provide strong evidence for ongoing local transmission in Ecuador and provide the first baseline characterization of *P. falciparum* antigenic diversity and parasite *var*codes circulating in the Ecuadorian northwest coast and Amazon regions. This forms a historical database that can be leveraged in future molecular epidemiology studies. It is worth noting that *var*code5 identified in Orellana province, an area of the Amazon region that neighbors Peru, had a very different genetic profile to the other Ecuadorian *var*codes (≥83% unique types). It is more genetically related to historical parasites from French Guiana and Venezuela pointing to highly diverse *P. falciparum* populations that are likely circulating in the Ecuadorian Amazon region and Ecuador-Peru border. Several recent outbreaks have occurred in the same border areas of Ecuador [in 2016 (9), 2018 (10), 2019 (11) and 2020 (62)]. Our findings of highly related and possible recombinant parasites of the outbreak clone causing clinical disease shortly after the outbreak may provide an explanation for how sustained epidemics of disease continue to occur locally. The importation of parasites combined with local transmission can clearly increase the pool of antigenic variants as well as overall genome diversity, although periodic outbreaks of local parasites may also provide sufficient conditions for this to occur locally in the absence of parasite importation.

We demonstrated that 58% of clinical cases sampled in 2014 immediately after the outbreak in Ecuador were caused by parasites with highly related or possibly recombinant *var*codes. The same trend was observed in 2015, although our sampling was limited relative to the number of reported cases for the months sampled. Is this a chance finding, or a consequence of selection, i.e., drug or immune selection? Previously published drug-resistance marker genotyping data reported that all *P. falciparum* isolates in our study had genotypes associated with chloroquine-resistance (i.e., *pfcrt* 76T) and the majority had genotypes associated with sensitivity to sulfadoxine-pyrimethamine (i.e., *pfdhps and pfdhfr*) (36). Mutations in *pfdhfr* were only present in parasites with *var*code2 and one of the highly related *var*codes (*var*code6) but not the other *var*codes. Past antimalarial treatment for *P. falciparum* malaria in Ecuador included artesunate and sulfadoxine-pyrimethamine alone or in combination with primaquine until 2012 when a switch to artemether-lumefantrine +primaquine was introduced as the first-line treatment (63). Thus, it does not appear from our findings that drug selection is associated with the *var* population structure we observe in clinical cases after the outbreak. Therefore, we hypothesize that parasites with these highly related *var*codes have new or antigenically novel combinations of *var* DBLα types and possibly alleles of single copy diverse antigen-encoding genes. This may provide an advantage in a population with variable levels of immune memory, as would be expected for these communities in Ecuador where seroprevalence of antibodies against *P. falciparum* has been reported to be as high as 22% (12). Supporting this view, prior network analyses and computational models combining evolution and epidemiology point to variant-specific immune selection defining *var* population structure even in low transmission, albeit to a much lesser extent than expected in high-transmission settings where selection acts strongly against recombinant or highly related repertoires due to cross-immunity (37, 64). The availability of *var* DBLα sequences provides the potential to test this hypothesis by serological methods that measure variant-specific immunity, as shown in serological studies in Papua New Guinea (65) and the Brazilian Amazon (66). In addition, characterization of antigenic variant *var* DBLα sequences could be leveraged in future gene expression studies to understand parasite virulence. Indeed, future investigations into a potential fitness advantage of *var*code1 and how it may enhance malaria transmission are warranted, in addition to exploring the origins of more recent outbreaks and whether they are the same or new *var*codes.

A previous study that included some of the same *P. falciparum* isolates from Esmeraldas City and San Lorenzo in the northwest coast described three main genetic clusters based on microsatellite genotyping (35). In contrast, when considering the same isolates, *var*coding resolved six *var*codes circulating. Similar observations have been described in an earlier study in Venezuela where sympatric parasites identical at neutral microsatellite loci were shown to have very different *var* repertoires (67). This is not surprising as *var*coding is expected to provide higher resolution than microsatellite genotyping as we are looking at more loci, which are under selection. Importantly, our *var*code membership designations are consistent with genomic lineages as defined by identity-by-descent (IBD) analyses (IBD ≥0.99) and “super-clusters” (IBD ≥0.80) from a recent whole genome sequencing (WGS) study of *P. falciparum* isolates from the Pacific Coast of Ecuador and Colombia collected in the early 2000s and in 2013–2017 (including 30 of the same isolates in this study) (56). Although *P. falciparum* WGS data exclude *var* data due to the difficulty of assembling highly diverse variant antigen genes, the WGS results are consistent with the population structure we infer from *var*coding. To our knowledge this is the first direct comparison of parasite relatedness inference as determined by WGS IBD and *var*coding P_TS_ (and BP_TS_) where, given the low transmission in this setting, defining population structure by neutral markers and *var*coding appears to converge. This provides compelling evidence of the utility of *var*coding and downstream P_TS_ and BP_TS_ analytical approaches in such settings. Indeed, it is noteworthy that *var*coding predicted the same genomic lineages and resolved parasite relatedness in line with WGS analyses, with the added advantage of simultaneously providing surveillance information about the major variant surface antigen-encoding genes. An interesting line of inquiry beyond the scope of this study will be to explore whether IBD-based approaches can be applied to *var*coding data, particularly in elimination settings where the population may be highly inbred and most infections are monoclonal.

Amplicon sequencing is being promoted as a potential cost-effective approach for molecular surveillance to inform on transmission patterns, potential parasite importation and parasite relatedness for malaria elimination efforts. *Var*coding is a targeted amplicon sequencing approach requiring a single PCR with degenerate primers to amplify 40–60 genes from the *var* multigene family encoding the most immunogenic protein family of *P. falciparum*. Here we demonstrate its potential as a cost effective and high-resolution method to examine *P. falciparum* antigenic diversity, parasite relatedness and genome-wide diversity patterns for molecular surveillance in low-transmission settings where highly related parasites are circulating. This will prove particularly useful in the context of changing patterns of human mobility and gene flow in the Americas where there is high demand for such a molecular surveillance method. Given that malaria elimination is achieved locally, *var*coding may be useful in areas where WGS may not be conducted routinely, or where infrastructure and local capacity may not exist. Even in areas where WGS is routinely conducted, *var*coding has the potential for screening samples and identifying the need for downstream WGS to save on resources. The tool will also be useful in other epidemic or low-transmission settings targeting elimination across the globe. Undoubtedly, going the distance to elimination by 2025 must be supported by appropriate molecular surveillance to better understand and track disease transmission as well as uncover the existence of local reservoirs of antigenically diverse parasites.

## Supplementary Material

Ruybal et al Supp

## Figures and Tables

**TABLE 1:** Epidemiological characteristics of study participants.

Case	Month collected		Age range, years	Sex	Sampling location (County, Province)	Self-reported infection location*	*var*code	Putative origin**
**EC1**	Jan 2013	*Outbreak*	NA	NA	Esmeraldas City, Esmeraldas		varcode1	Local
**EC2**	Jan 2013	*Outbreak*	NA	NA	Esmeraldas City, Esmeraldas		varcode1	Local
**EC3**	May 2013	*Outbreak*	>30–40	M	Esmeraldas City, Esmeraldas		varcode1	Local
**EC4**	May 2013	*Outbreak*	>30–40	M	Esmeraldas City, Esmeraldas		varcode1	Local
**EC5**	May 2013	*Outbreak*	>50–60	F	Esmeraldas City, Esmeraldas		varcode1	Local
**EC6**	May 2013	*Outbreak*	>30–40	M	Esmeraldas City, Esmeraldas		varcode1	Local
**EC7**	May 2013	*Outbreak*	>50–60	F	Esmeraldas City, Esmeraldas		varcode1	Local
**EC8**	May 2013	*Outbreak*	>10–20	F	Esmeraldas City, Esmeraldas		varcode1	Local
**EC9**	May 2013	*Outbreak*	>10–20	F	Esmeraldas City, Esmeraldas		varcode1	Local
**EC10**	May 2013	*Outbreak*	>10–20	M	Esmeraldas City, Esmeraldas		varcode1	Local
**EC11**	May 2013	*Outbreak*	>20–30	F	Esmeraldas City, Esmeraldas		varcode1	Local
**EC12**	Jun 2013	*Outbreak*	>10–20	F	Esmeraldas City, Esmeraldas		varcode1	Local
**EC13**	Jun 2013	*Outbreak*	>10–20	M	Esmeraldas City, Esmeraldas		varcode1	Local
**EC14**	Jun 2013	*Outbreak*	>40–50	M	Esmeraldas City, Esmeraldas		varcode1	Local
**EC15**	Jun 2013	*Outbreak*	>40–50	M	Esmeraldas City, Esmeraldas		varcode1	Local
**EC16**	Jun 2013	*Outbreak*	>10–20	F	Esmeraldas City, Esmeraldas		varcode1	Local
**EC17**	Jun 2013	*Outbreak*	>10–20	F	Esmeraldas City, Esmeraldas		varcode1	Local
**EC18**	Jun 2013	*Outbreak*	>20–30	M	Esmeraldas City, Esmeraldas		varcode1	Local
**EC19**	Jun 2013	*Outbreak*	>40–50	M	Esmeraldas City, Esmeraldas		varcode1	Local
**EC20**	Jul 2013		>20–30	M	San Lorenzo, Esmeraldas		varcode2	Local
**EC21**	Jul 2013		>10–20	F	San Lorenzo, Esmeraldas		varcode2	Local
**EC22**	Jul 2013	*Outbreak*	>10–20	M	Esmeraldas City, Esmeraldas		varcode1	Local
**EC23**	Jul 2013	*Outbreak*	>0–10	M	Esmeraldas City, Esmeraldas		varcode1	Local
**EC24**	Jul 2013	*Outbreak*	NA	F	Esmeraldas City, Esmeraldas		varcode1	Local
**EC25**	Sep 2013	*Outbreak*	>30–40	M	Esmeraldas City, Esmeraldas		varcode1	Local
**EC26**	Oct 2013	*Outbreak*	>20–30	F	Esmeraldas City, Esmeraldas		varcode1	Local
**EC27**	Oct 2013		>10–20	F	San Lorenzo, Esmeraldas		varcode3	Local
**EC28**	Oct 2013	*Outbreak*	>20–30	M	Esmeraldas City, Esmeraldas		varcode1	Local
**EC29**	Nov 2013	*Outbreak*	>10–20	M	Esmeraldas City, Esmeraldas		varcode1	Local
**EC30**	Nov 2013	*Outbreak*	>10–20	M	Esmeraldas City, Esmeraldas		varcode1	Local
**EC31**	Nov 2013	*Outbreak*	>10–20	F	Esmeraldas City, Esmeraldas		varcode1	Local
**EC32**	Nov 2013	*Outbreak*	>30–40	M	Esmeraldas City, Esmeraldas		varcode1	Local
**EC33**	Nov 2013	*Outbreak*	>30–40	M	Esmeraldas City, Esmeraldas		varcode1	Local
**EC34**	Jan 2014		NA	NA	Jaramijo, Manabi		varcode3	Local
**EC35**	Jan 2014		>50–60	F	San Lorenzo, Esmeraldas		varcode1	Local
**EC36**	Jan 2014		>10–20	M	San Lorenzo, Esmeraldas		varcode1	Local
**EC37**	Jan 2014		>30–40	M	San Lorenzo, Esmeraldas		varcode2	Local
**EC38**	Jan 2014		>10–20	M	San Lorenzo, Esmeraldas		varcode4	Local
**EC39**	Jan 2014		>10–20	M	San Lorenzo, Esmeraldas		varcode1	Local
**EC40**	Jan 2014		>30–40	F	Nuevo Rocafuerte, Orellana	Peru	varcode5	French Guiana/Peru/Venezuela
**EC41**	Mar 2014		>20–30	M	San Lorenzo, Esmeraldas		varcode6	Local
**EC42**	May 2014		>50–60	F	Esmeraldas City, Esmeraldas	Colombia	varcode3	Local
**EC43**	May 2014		NA	NA	Cascales, Sucumbios	San Lorenzo, Esmeraldas	varcode1	Local
**EC44**	Jul 2014		>30–40	F	San Lorenzo, Esmeraldas		varcode7	Local
**EC45**	Aug 2014		>20–30	F	San Lorenzo, Esmeraldas		varcode7	Local
**EC46**	Aug 2014		>20–30	M	San Lorenzo, Esmeraldas		varcode7	Local
**EC47**	Aug 2014		>50–60	M	San Lorenzo, Esmeraldas		varcode7	Local
**EC48**	Aug 2014		>20–30	M	San Lorenzo, Esmeraldas		varcode7	Local
**EC49**	Sep 2014		NA	F	Lago Agrio, Sucumbios	Colombia	varcode8	Local
**EC50**	Oct 2014		NA	NA	Lago Agrio, Sucumbios	Colombia	varcode8	Local
**EC51**	Oct 2014		>30–40	M	San Lorenzo, Esmeraldas	Colombia	varcode6	Local
**EC52**	Dec 2014		>20–30	M	San Lorenzo, Esmeraldas		varcode7	Local
**EC53**	Jan 2015		NA	NA	Lumbaqui, Sucumbios	Colombia	varcode9	Colombia
**EC54**	Jan 2015		>20–30	M	San Lorenzo, Esmeraldas		varcode7	Local
**EC55**	Jan 2015		>10–20	M	San Lorenzo, Esmeraldas		varcode7	Local
**EC56**	Nov 2015		>10–20	F	San Lorenzo, Esmeraldas		varcode6	Local
**EC57**	May 2015		NA	NA	Tobar Donoso, Carchi		varcode1	Local
**EC58**	May 2015		NA	NA	Tobar Donoso, Carchi		varcode1	Local

NA refers to no data collected for that particular epidemiological variable.

* The self-reported infection location was recorded at the time of sample collection based on the answers provided by the study participants.

** The putative origin as determined by *var*coding.

## Data Availability

The datasets presented in this study can be found in online repositores. The cleaned DBLɑ sequences generated in this study have been submitted to DDBJ/ENA/GenBank (BioProject Number: PRJNA642683). The python scripts for DBLɑ sequence processing can be found on GitHub in the following open-source repositories: DBLɑCleaner pipeline (https://github.com/Unimelb-Day-Lab/DBLaCleaner); clusterDBLalpha pipeline (https://github.com/Unimelb-Day-Lab/clusterDBLalpha); classifyDBLalpha pipeline (https://github.com/Unimelb-Day-Lab/classifyDBLalpha) with a tutorial detailing the data processing workflow at https://github.com/Unimelb-Day-Lab/tutorialDBLalpha. All other deidentified data and analysis code are available on the open-source GitHub repository at https://github.com/shaziaruybal/varcode-ecuador.

## References

[1] Organization WH. Eliminating malaria. Geneva: World Health Organization (2016). Available at: https://www.who.int/malaria/publications/atoz/eliminating-malaria/en/.

[2] Organization WH. Zeroing in on malaria elimination: Final report of the e-2020 initiative. Geneva: World Health Organization (2021). Available at: https://apps.who.int/iris/bitstream/handle/10665/340881/9789240024359-eng.pdf.

[3] Espinoza J Malaria resurgence in the americas: An underestimated threat. Pathogens (2019) 8:11. doi: 10.3390/pathogens8010011 30669301 PMC6471461

[4] Jaramillo-Ochoa R, Sippy R, Farrell DF, Cueva-Aponte C, Beltrán-Ayala E, Gonzaga JL, Effects of political instability in Venezuela on malaria resurgence at Ecuador–Peru border, 2018. Emerg Infect Dis (2019) 25:834–6. doi: 10.3201/eid2504.181355 30698522 PMC6433012

[5] Ferreira MU, Castro MC. Malaria situation in Latin America and the Caribbean: Residual and resurgent transmission and challenges for control and elimination. Methods Mol Biol Clifton N J (2019) 2013:57–70. doi: 10.1007/978-1-4939-9550-9_4 31267493

[6] Organization WH. World malaria report 2019. Geneva: World Health Organization (2019).

[7] Organization WH. The e-2020 initiative of 21 malaria-eliminating countries: 2019 progress report. Geneva: World Health Organization (2019).

[8] MSP. Gaceta epidemiológica semanal no. 52. Quito: Ministerio de Salud Pública del Ecuador (2016).

[9] MSP. Gaceta epidemiológica semanal no. 52. Quito: Ministerio de Salud Pública del Ecuador (2017).

[10] MSP. Gaceta epidemiológica semanal no. 52. Quito: Ministerio de Salud Pública del Ecuador (2018).

[11] MSP. Gaceta epidemiológica semanal no. 52. Quito: Ministerio de Salud Pública del Ecuador (2019).

[12] Sáenz FE, Arévalo-Cortés A, Valenzuela G, Vallejo AF, Castellanos A, Poveda-Loayza AC, Malaria epidemiology in low-endemicity areas of the northern coast of Ecuador: high prevalence of asymptomatic infections. Malaria J (2017) 16:300. doi: 10.1186/s12936-017-1947-0 PMC5530496 28747199

[13] Griffing SM, Mixson-Hayden T, Sridaran S, Alam MT, McCollum AM, Cabezas C, South American plasmodium falciparum after the malaria eradication era: Clonal population expansion and survival of the fittest hybrids. PloS One (2011) 6: e23486. doi: 10.1371/journal.pone.0023486 21949680 PMC3174945

[14] Larrañaga N, Mejıá RE, Hormaza JI, Montoya A, Soto A, Fontecha GA. Genetic structure of plasmodium falciparum populations across the Honduras-Nicaragua border. Malaria J (2013) 12:354. doi: 10.1186/1475-2875-12-354 PMC3851272 24093629

[15] Obaldia N, Baro NK, Calzada JE, Santamaria AM, Daniels R, Wong W, Clonal outbreak of plasmodium falciparum infection in eastern Panama. J Infect Dis (2014) 211:1087–96. doi: 10.1093/infdis/jiu575 25336725 PMC4366603

[16] Okoth SA, Chenet SM, Arrospide N, Gutierrez S, Cabezas C, Matta JA, Molecular investigation into a malaria outbreak in cusco, Peru: Plasmodium falciparum BV1 lineage is linked to a second outbreak in recent times. Am J Trop Med Hyg (2015) 94:128–31. doi: 10.4269/ajtmh.15-0442 26483121 PMC4710416

[17] Baldeviano GC, Okoth SA, Arrospide N, Gonzalez RV, Sánchez JF, Macedo S, Molecular epidemiology of plasmodium falciparum malaria outbreak, tumbes, Peru, 2010–2012. Emerg Infect Dis (2015) 21:797–803. doi: 10.3201/eid2105.141427 25897626 PMC4412223

[18] Chenet SM, Taylor JE, Blair S, Zuluaga L, Escalante AA. Longitudinal analysis of plasmodium falciparum genetic variation in turbo, Colombia: implications for malaria control and elimination. Malaria J (2015) 14:363. doi: 10.1186/s12936-015-0887-9 PMC4578328 26395166

[19] Sáenz FE, Morton LC, Okoth SA, Valenzuela G, Vera-Arias CA, Vélez-Álvarez E, Clonal population expansion in an outbreak of plasmodium falciparum on the northwest coast of Ecuador. Malaria J (2015) 14:497. doi: 10.1186/s12936-015-1019-2 PMC4676133 26651993

[20] Carter TE, Malloy H, Existe A, Memnon G, Victor YS, Okech BA, Genetic diversity of plasmodium falciparum in Haiti: Insights from microsatellite markers. PLoS One (2015) 10:e0140416. doi: 10.1371/journal.pone.0140416 26462203 PMC4604141

[21] Dalmat R, Naughton B, Kwan-Gett TS, Slyker J, Stuckey EM. Use cases for genetic epidemiology in malaria elimination. Malaria J (2019) 18:163. doi: 10.1186/s12936-019-2784-0 PMC6503548 31064369

[22] Neafsey DE, Taylor AR, MacInnis BL. Advances and opportunities in malaria population genomics. Nat Rev Genet (2021) 22:502–17. doi: 10.1038/s41576-021-00349-5 33833443 PMC8028584

[23] Grenfell BT, Pybus OG, Gog JR, Wood JLN, Daly JM, Mumford JA, Unifying the epidemiological and evolutionary dynamics of pathogens. Science (2004) 303:327–32. doi: 10.1126/science.1090727 14726583

[24] Volz EM, Koelle K, Bedford T. Viral phylodynamics. PLoS Comput Biol (2013) 9:e1002947. doi: 10.1371/journal.pcbi.1002947 23555203 PMC3605911

[25] Barton N Understanding adaptation in Large populations. PLoS Genet (2010) 6: e1000987. doi: 10.1371/journal.pgen.1000987 20585547 PMC2887463

[26] Rask TS, Hansen DA, Theander TG, Pedersen AG, Lavstsen T. Plasmodium falciparum erythrocyte membrane protein 1 diversity in seven genomes – divide and conquer. PLoS Comput Biol (2010) 6:e1000933. doi: 10.1371/journal.pcbi.1000933 20862303 PMC2940729

[27] Otto TD, Assefa SA, Böhme U, Sanders MJ, Kwiatkowski Dconsortium P, Evolutionary analysis of the most polymorphic gene family in falciparum malaria. Wellcome Open Res (2019) 4:193. doi: 10.12688/wellcomeopenres.15590.1 32055709 PMC7001760

[28] Barry AE, Leliwa-Sytek A, Tavul L, Imrie H, Migot-Nabias F, Brown SM, Population genomics of the immune evasion (var) genes of plasmodium falciparum. PLoS Pathog (2007) 3:e34. doi: 10.1371/journal.ppat.0030034 17367208 PMC1828697

[29] Chen DS, Barry AE, Leliwa-Sytek A, Smith T-A, Peterson I, Brown SM, A molecular epidemiological study of var gene diversity to characterize the reservoir of plasmodium falciparum in humans in Africa. PLoS One (2011) 6:e16629. doi: 10.1371/journal.pone.0016629 21347415 PMC3036650

[30] Rougeron V, Tiedje KE, Chen DS, Rask TS, Gamboa D, Maestre A, Evolutionary structure of plasmodium falciparum major variant surface antigen genes in south America: Implications for epidemic transmission and surveillance. Ecol Evol (2017) 7:9376–90. doi: 10.1002/ece3.3425 29187975 PMC5696401

[31] Tonkin-Hill G, Ruybal-Pesántez S, Tiedje KE, Rougeron V, Duffy MF, Zakeri S, Evolutionary analyses of the major variant surface antigen-encoding genes reveal population structure of plasmodium falciparum within and between continents. PloS Genet (2021) 17:e1009269. doi: 10.1371/journal.pgen.1009269 33630855 PMC7906310

[32] Day KP, Artzy-Randrup Y, Tiedje KE, Rougeron V, Chen DS, Rask TS, Evidence of strain structure in plasmodium falciparum var gene repertoires in children from Gabon, West Africa. Proc Natl Acad Sci (2017) 114:E4103–11. doi: 10.1073/pnas.1613018114 28461509 PMC5441825

[33] Ruybal-Pesántez S, Tiedje KE, Tonkin-Hill G, Rask TS, Kamya MR, Greenhouse B, Population genomics of virulence genes of plasmodium falciparum in clinical isolates from Uganda. Sci Rep-uk (2017) 7:11810. doi: 10.1038/s41598-017-11814-9 PMC5603532 28924231

[34] Lucchi NW, Narayanan J, Karell MA, Xayavong M, Kariuki S, DaSilva AJ, Molecular diagnosis of malaria by photo-induced electron transfer fluorogenic primers: PET-PCR. PLoS One (2013) 8:e56677. doi: 10.1371/journal.pone.0056677 23437209 PMC3577666

[35] Vera-Arias CA, Castro LE, Gómez-Obando J, Sáenz FE. Diverse origin of plasmodium falciparum in northwest Ecuador. Malaria J (2019) 18:251. doi: 10.1186/s12936-019-2891-y PMC6660669 31349843

[36] Valenzuela G, Castro LE, Valencia-Zamora J, Vera-Arias CA, Rohrbach P, Sáenz FE. Genotypes and phenotypes of resistance in Ecuadorian plasmodium falciparum. Malaria J (2019) 18:415. doi: 10.1186/s12936-019-3044-z PMC6905098 31822269

[37] He Q, Pilosof S, Tiedje KE, Ruybal-Pesántez S, Artzy-Randrup Y, Baskerville EB, Networks of genetic similarity reveal non-neutral processes shape strain structure in plasmodium falciparum. Nat Commun (2018) 9:1817. doi: 10.1038/s41467-018-04219-3 29739937 PMC5940794

[38] Taylor HM, Kyes SA, Harris D, Kriek N, Newbold CI. A study of var gene transcription *in vitro* using universal var gene primers. Mol Biochem Parasit (2000) 105:13–23. doi: 10.1016/s0166-6851(99)00159-0 10613695

[39] Roche. Using multiplex identifier (MID) adaptors for the GS FLX titanium chemistry–extended MID set (2009). Available at: https://dna.uga.edu/wp-content/uploads/sites/51/2013/12/GS-FLX-Titanium-General-Library-Preparation-Method-Manual-Roche.pdf.

[40] Ruybal-Pesántez S, Tiedje KE, Piloso S, Tonkin-Hill G, He Q, Rask TS, Age-specific patterns of DBLα var diversity can explain why residents of high malaria transmission areas remain susceptible to plasmodium falciparum blood stage infection throughout life. Int J Parasitol (2021) 52(11):721–31. doi: 10.1016/j.ijpara.2021.12.001 PMC9339046 35093396

[41] Oksanen J, Blanchet FG, Friendly M, Kindt R, Legendre P, McGlinn D, Vegan: Community ecology package (2019). Available at: https://CRAN.R-project.org/package=vegan.

[42] Larremore DB. Bayes-optimal estimation of overlap between populations of fixed size. PLoS Comput Biol (2019) 15:e1006898. doi: 10.1371/journal.pcbi.1006898 30925165 PMC6440621

[43] Johnson EK, Larremore DB. Bayesian Estimation of community size and overlap from random subsamples. PLoS Comput Biol (2022) 18:e1010451. doi: 10.1371/journal.pcbi.1010451 36121879 PMC9522272

[44] Otto TD, Böhme U, Sanders M, Reid A, Bruske EI, Duffy CW, Long read assemblies of geographically dispersed plasmodium falciparum isolates reveal highly structured subtelomeres. Wellcome Open Res (2018) 3:52. doi: 10.12688/wellcomeopenres.14571.1 29862326 PMC5964635

[45] Pedersen TL. Ggraph: An implementation of grammar of graphics for graphs and networks (2020). Available at: https://CRAN.R-project.org/package=ggraph.

[46] Pedersen TL. Tidygraph: A tidy API for graph manipulation. (2019). Available at: https://CRAN.R-project.org/package=tidygraph.

[47] Dunnington D Ggspatial: Spatial data framework for ggplot2 (2018). Available at: https://CRAN.R-project.org/package=ggspatial.

[48] Pedersen TL, Robinson D. Gganimate: A grammar of animated graphics (2020). Available at: https://CRAN.R-project.org/package=gganimate.

[49] Kolde R Pheatmap: Pretty heatmaps (2019). Available at: https://CRAN.R-project.org/package=pheatmap.

[50] Paradis E, Schliep K. Ape 5.0: an environment for modern phylogenetics and evolutionary analyses in r. Bioinformatics (2018) 35:526–8. doi: 10.1093/bioinformatics/bty633 30016406

[51] Yu G, Smith DK, Zhu H, Guan Y, Lam TT. ggtree : an r package for visualization and annotation of phylogenetic trees with their covariates and other associated data. Methods Ecol Evol (2016) 8:28–36. doi: 10.1111/2041-210x.12628

[52] Yu G, Lam TT-Y, Zhu H, Guan Y. Two methods for mapping and visualizing associated data on phylogeny using ggtree. Mol Biol Evol (2018) 35:3041–3. doi: 10.1093/molbev/msy194 30351396 PMC6278858

[53] Team RC. R: A language and environment for statistical computing. Vienna, Austria: R Foundation for Statistical Computing (2019). Available at: https://www.R-project.org/.

[54] Wickham H, François R, Henry L, Müller K. Dplyr: A grammar of data manipulation (2020). Available at: https://CRAN.R-project.org/package=dplyr.

[55] Stevenson M epiR: Tools for the analysis of epidemiological data (2020). Available at: https://CRAN.R-project.org/package=epiR.

[56] Carrasquilla M, Early AM, Taylor AR, Ospina AK, Echeverry DF, Anderson TJC, Resolving drug selection and migration in an inbred south American plasmodium falciparum population with identity-by-descent analysis. PLoS Pathog (2022) 18:e1010993. doi: 10.1371/journal.ppat.1010993 36542676 PMC9815574

[57] Branch OH, Sutton PL, Barnes C, Castro JC, Hussin J, Awadalla P, Plasmodium falciparum genetic diversity maintained and amplified over 5 years of a low transmission endemic in the Peruvian Amazon. Mol Biol Evol (2010) 28:1973–86. doi: 10.1093/molbev/msq311 21109587 PMC3112368

[58] Echeverry DF, Nair S, Osorio L, Menon S, Murillo C, Anderson TJ. Long term persistence of clonal malaria parasite plasmodium falciparum lineages in the Colombian pacific region. BMC Genet (2013) 14:2. doi: 10.1186/1471-2156-14-2 23294725 PMC3563461

[59] Douine M, Mosnier E, Hingrat QL, Charpentier C, Corlin F, Hureau L, Illegal gold miners in French Guiana: a neglected population with poor health. BMC Public Health (2017) 18:23. doi: 10.1186/s12889-017-4557-4 28716015 PMC5513330

[60] Douine M, Sanna A, Hiwat H, Briolant S, Nacher M, Belleoud D, Investigation of a possible malaria epidemic in an illegal gold mine in French Guiana: an original approach in the remote Amazonian forest. Malaria J (2019) 18:91. doi: 10.1186/s12936-019-2721-2 PMC6431065 30902054

[61] Mosquera-Romero M, Zuluaga-Idárraga L, Tobón-Castaño A. Challenges for the diagnosis and treatment of malaria in low transmission settings in San Lorenzo, esmeraldas, Ecuador. Malaria J (2018) 17:440. doi: 10.1186/s12936-018-2591-z PMC6264637 30486839

[62] MSP. Personal communication. Ministerio de Salúd Pública (2020).

[63] Aguilar-Velasco HM. Malaria y espacio en el Ecuador del verde de parís a la eliminación de la enfermedad. Quito, Ecuador: Universidad Andina Simón Bolıvar, Sede Ecuador (2021). Available at: http://hdl.handle.net/10644/8216.

[64] Pilosof S, He Q, Tiedje KE, Ruybal-Pesántez S, Day KP, Pascual M. Competition for hosts modulates vast antigenic diversity to generate persistent strain structure in plasmodium falciparum. PLoS Biol (2019) 17:e3000336. doi: 10.1371/journal.pbio.3000336 31233490 PMC6611651

[65] Barry AE, Trieu A, Fowkes FJI, Pablo J, Kalantari-Dehaghi M, Jasinskas A, The stability and complexity of antibody responses to the major surface antigen of plasmodium falciparum are associated with age in a malaria endemic area. Mol Cell Proteomics (2011) 10:M111.008326. doi: 10.1074/mcp.m111.008326 PMC3226400 21825279

[66] Carlos BC, Fotoran WL, Menezes MJ, Cabral FJ, Bastos MF, Costa FTM, Expressed var gene repertoire and variant surface antigen diversity in a shrinking plasmodium falciparum population. Exp Parasitol (2016) 170:90–9. doi: 10.1016/j.exppara.2016.09.006 27663467

[67] Tami A, Ord R, Targett GA, Sutherland CJ. Sympatric plasmodium falciparum isolates from Venezuela have structured var gene repertoires. Malaria J (2003) 2:7. doi: 10.1186/1475-2875-2-7 PMC155546 12737636

