# Supplementary material for: Molecular epidemiology of continued Plasmodium falciparum disease transmission after an outbreak in Ecuador
Source: Front Trop Dis. Author manuscript; Available in PMC 2024 Nov 8. (PMC11546077; doi:10.3389/fitd.2023.1085862)
Supplement: Ruybal et al Supp [file NIHMS2022009-supplement-Ruybal_et_al_Supp.pdf]

Molecular epidemiology of continued *Plasmodium falciparum* disease transmission after an outbreak in Ecuador

Shazia Ruybal-Pesántez[1§*], Fabián E. Sáenz[2], Samantha L. Deed[1,3], Erik K. Johnson[4], Daniel B. Larremore[5,6], Claudia A. Vera-Arias[2#], Kathryn E. Tiedje[1,3], Karen P. Day[1,3*]

[1] School of BioSciences/Bio21 Institute, The University of Melbourne, Melbourne, Australia
[2] Centro de Investigación para la Salud en América Latina, Facultad de Ciencias Exactas y Naturales, Pontificia Universidad Católica del Ecuador, Av. 12 de octubre 1076, Apartado: 17-01-2184, Quito, Ecuador
[3] Department of Microbiology and Immunology, The University of Melbourne, Bio21 Institute and Peter Doherty Institute, Melbourne, Australia
[4] Department of Applied Mathematics, University of Colorado Boulder, Boulder, Colorado, USA
[5] Department of Computer Science, University of Colorado Boulder, Boulder, Colorado, USA
[6] BioFrontiers Institute, University of Colorado Boulder, Boulder, Colorado, USA

[§] *Current affiliations*: Department of Infectious Disease Epidemiology and MRC Centre for Global Infectious Disease Analysis, School of Public Health, Imperial College London, UK; Population Health and Immunity Division, Walter and Eliza Hall Institute of Medical Research, Melbourne, Australia; Instituto de Microbiología, Universidad San Francisco de Quito, Ecuador
[#] *Current affiliation*: Eck Institute for Global Health, University of Notre Dame, Notre Dame, IN, 46556, USA

*Correspondence:
Karen P. Day
karen.day@unimelb.edu.au

Shazia Ruybal-Pesántez
s.ruybal@imperial.ac.uk

Supplementary Figures

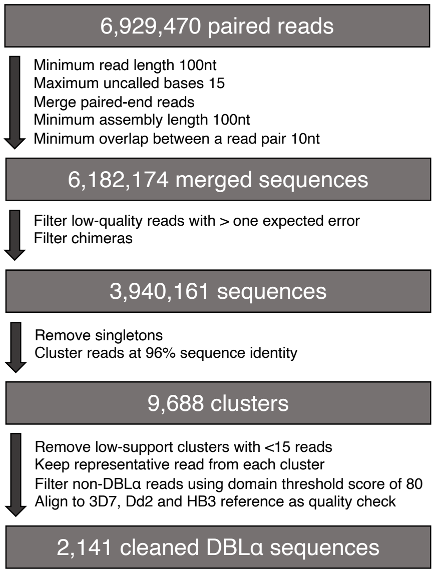

Figure S1. Bioinformatic sequence data processing flowchart. The flowchart shows the bioinformatic process to clean the raw de-multiplexed paired reads with details on the filtering parameters utilized at each step. This customized bioinformatic pipeline is described in more detail in He et al (1).

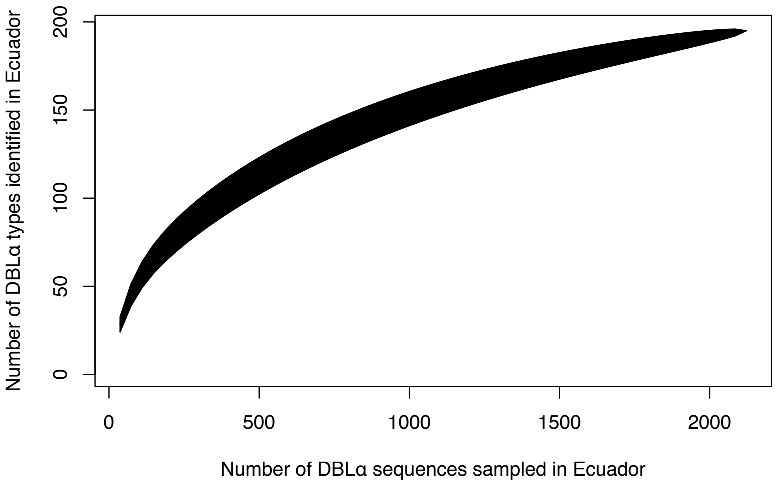

Figure S2. Sampling depth of *var* DBLα types in Ecuador. Sampling accumulation curves depict the number of observed DBLα types plotted as a function of the number of DBLα sequences sampled.

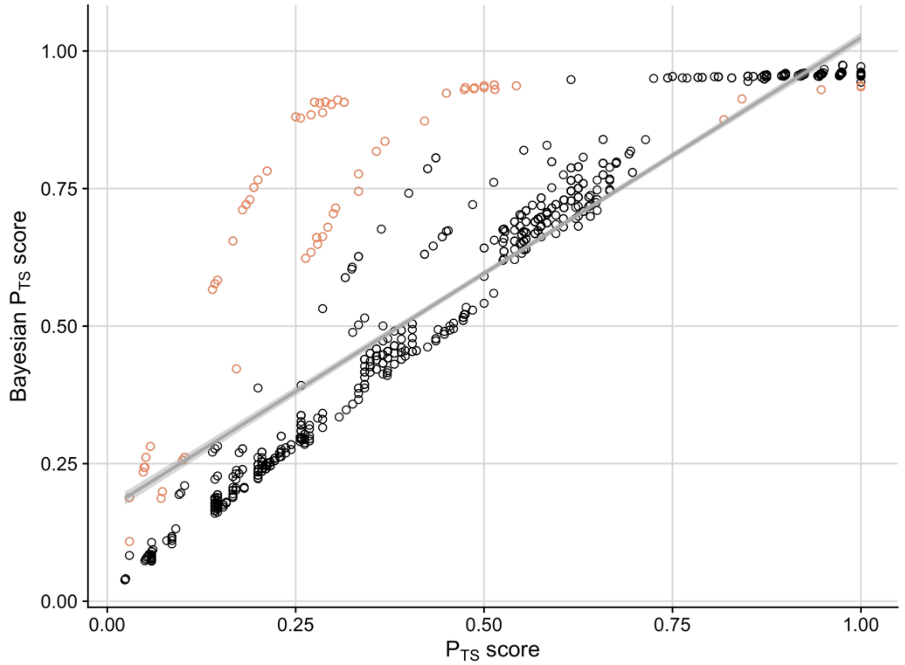

Figure S3. Relatedness of isolates based on observed and unbiased Bayesian pairwise type sharing estimates. There was a positive correlation between the two *var*code relatedness measures (correlation coefficient = 0.919, $p < 0.001$). Circles colored in light red correspond to pairwise comparisons involving the two *P. falciparum* isolates with the smallest repertoire sizes (11 and 19 DBLα types), many of which had lower statistical confidence and the largest discrepancies between observed and unbiased estimates, as expected.

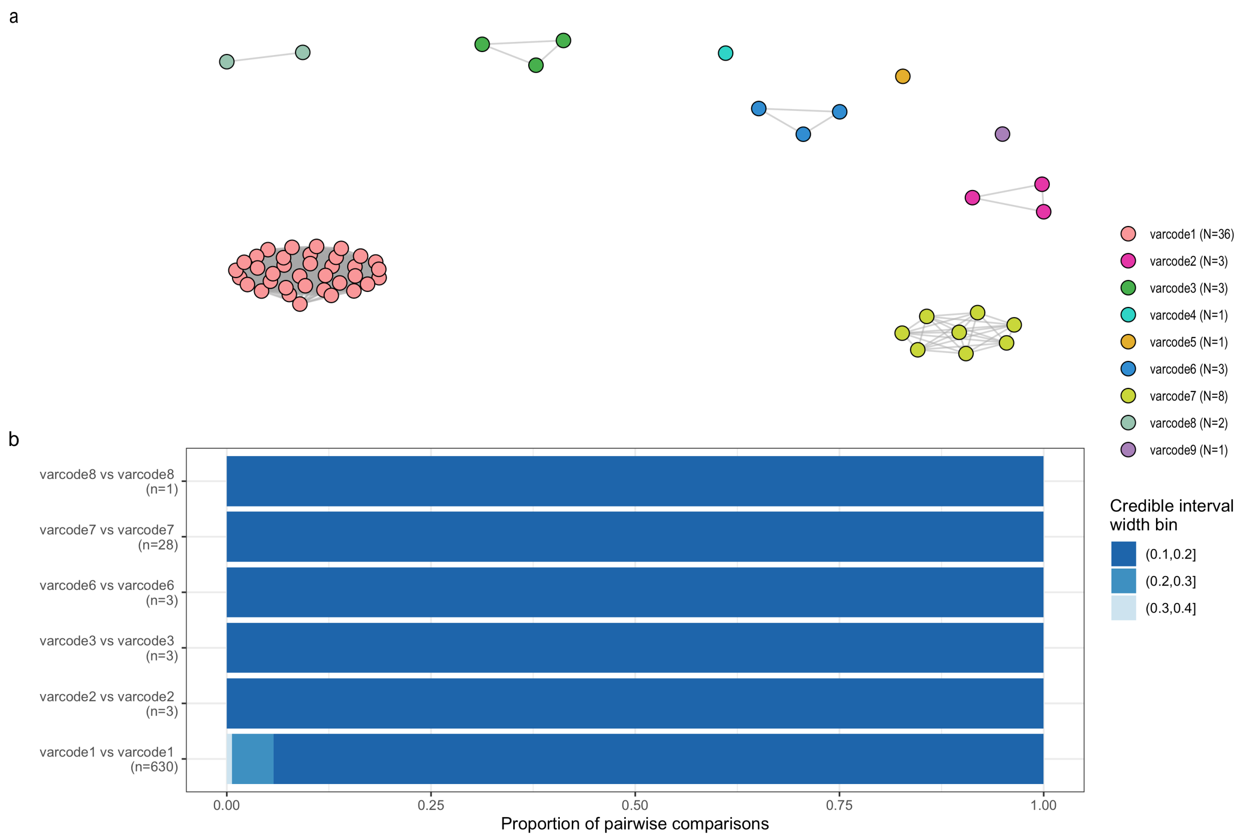

Figure S4. *Var*code relatedness networks based on Bayesian pairwise type sharing estimates to define *var*codes. A network visualization of the *var*code relatedness of *P. falciparum* isolates at the threshold of (a) BP_{TS} ≥0.90 to define clusters of genetically-related isolates based the mean posterior Bayesian PTS (BP_{TS}). Every node represents a *P. falciparum* isolate and an edge represents the BP_{TS} value between two particular nodes/isolates. Isolates that cluster together (i.e., connected by edges) are considered to be genetically identical (i.e., clones). Every color represents a different *var*code. (b) For each pairwise comparison, we calculated the highest density posterior interval (HDPI) width as an estimate of uncertainty (analogous to calculating the width of a frequentist confidence interval). The proportion of pairwise comparisons within a given 95% HDPI width are shown. There was high confidence (HDPI width ≤0.2) for 94.6% (632/668) of pairwise BP_{TS} estimates. Low confidence pairwise BP_{TS} (HDPI width >0.2; max = 0.33) were only found between the two *var*code1 isolates with the smallest repertoire sizes (11 and 19 DBLα types; 36/36 comparisons). Note: only *var*codes with >1 *P. falciparum* isolate are shown. For the full distribution of within-*var*code BP_{TS} estimates see Fig. S5b.

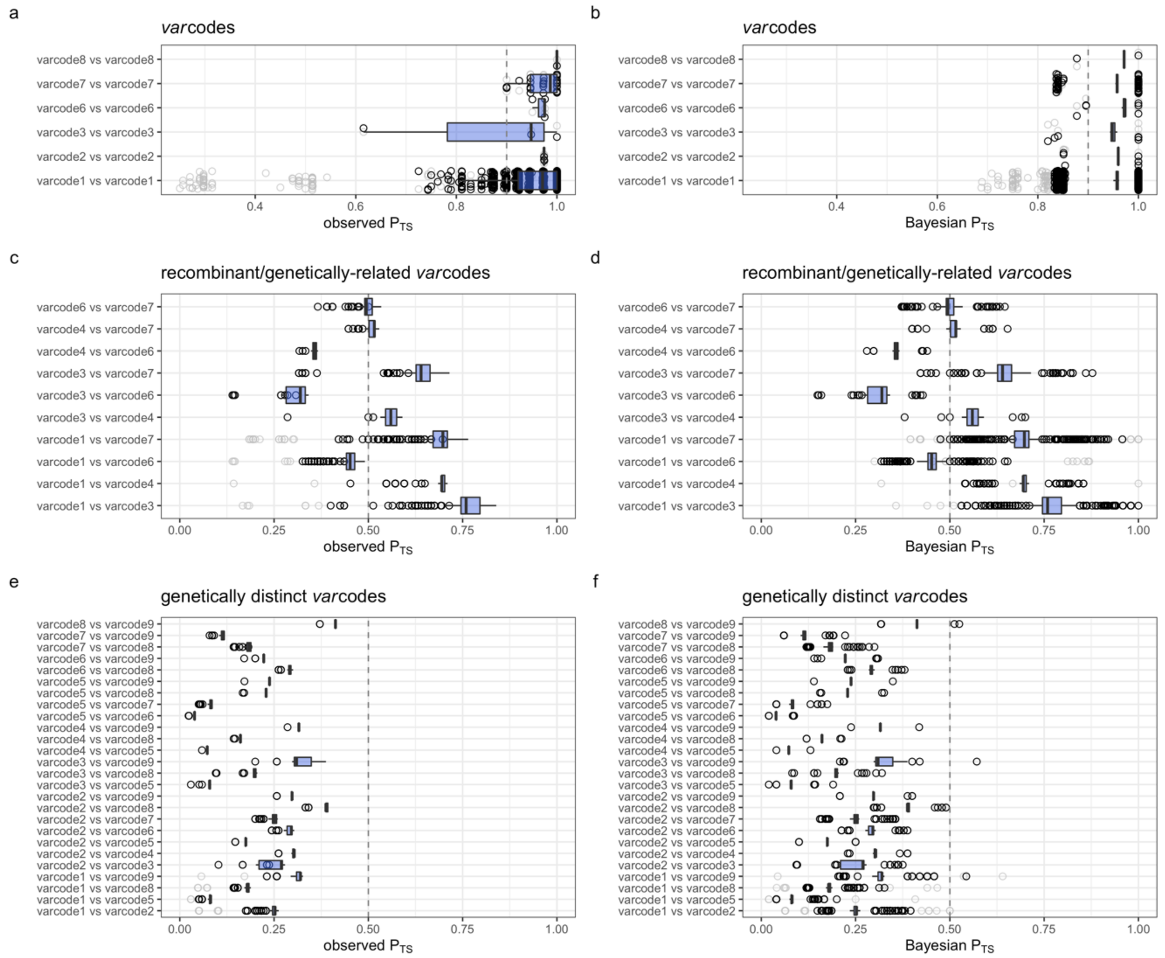

Figure S5. Distribution of observed and Bayesian pairwise type sharing estimates used to define *var*codes, as well as highly-related/recombinant or genetically-related, and genetically distinct *var*codes. For observed P_{TS} estimates (a, c, e), the boxplots (in blue) show the median and interquartile range of the observed P_{TS} estimates (circles) for all pairwise comparisons among *P. falciparum* isolates for a given within-*var*code or between-*var*code comparison. For Bayesian PTS (BP_{TS}) estimates (b, d, f), the boxplots (in blue) show the median and interquartile range of the posterior mean BP_{TS} estimates. The circles show the BP_{TS} estimates for the lower and upper bound of the 95% highest density posterior intervals (HDPIs). Circles showing all the posterior mean BP_{TS} estimates are omitted for clarity. Dashed lines indicate the threshold of (a-b) P_{TS} or BP_{TS} ≥0.90 and (c-f) P_{TS} or BP_{TS} ≥0.50. For comparison to the *var*code relatedness network edges see Figures 3a, 3c, S4a, S6a. In all panels, light grey circles correspond to pairwise comparisons involving the two *P. falciparum* isolates with the smallest repertoire sizes (11 and 19 DBLα types), many of which had lower statistical confidence and the largest discrepancies between observed and unbiased estimates, as expected. Black circles denote all other estimates. Only *var*codes with >1 *P. falciparum* isolate are shown. For the *var*codes that were highly-related/recombinants, 70.9% (433/611) of the HDPIs were confidently above the threshold of BP_{TS} ≥0.50 and for *var*codes that were genetically-distinct, 98.4% (368/374) of the HDPIs were confidently below the threshold of BP_{TS} <0.50.

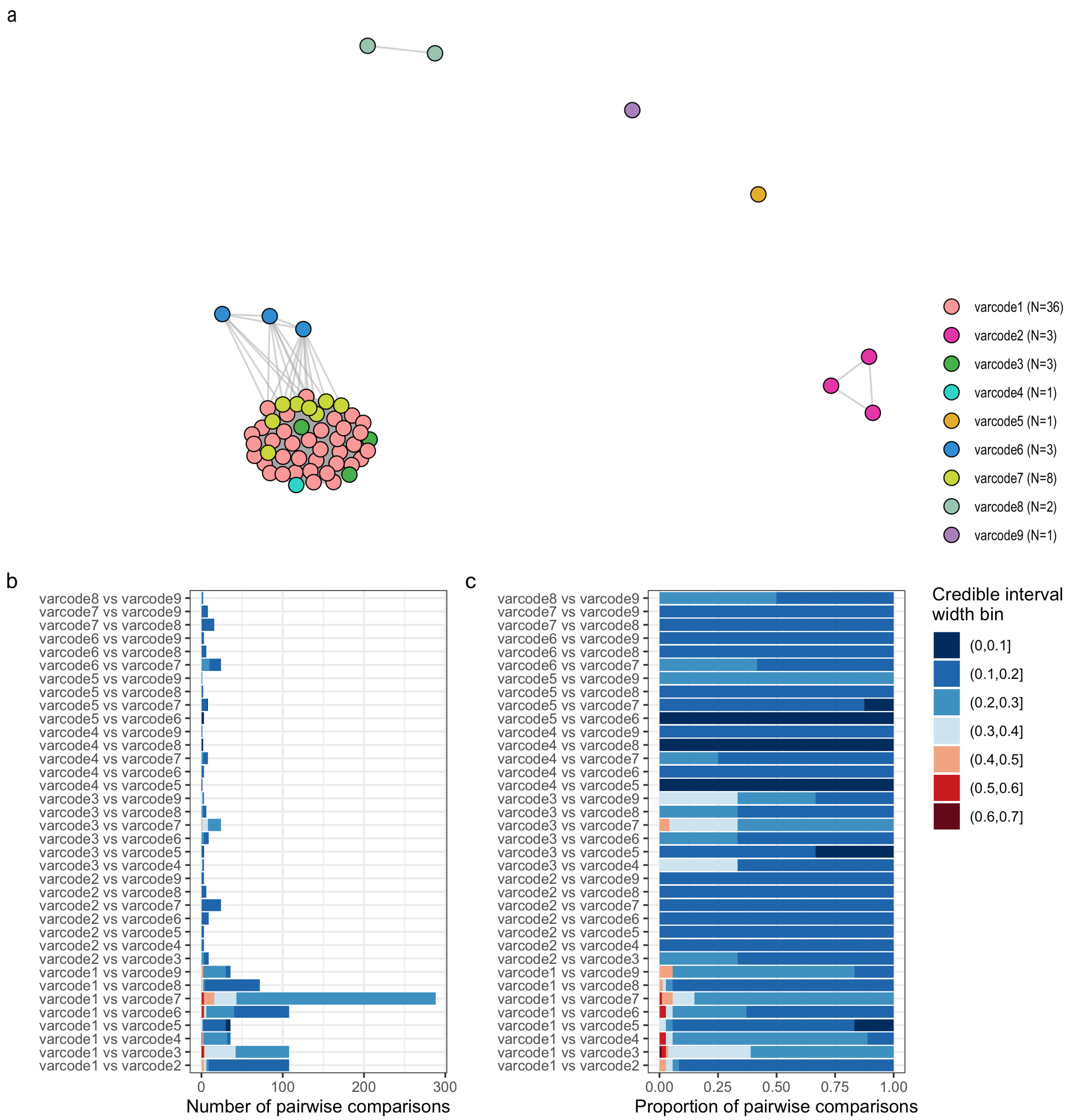

Figure S6. *Var*code relatedness networks based on unbiased Bayesian pairwise type sharing estimates to identify recombinants. A network visualization of the *var*code relatedness of *P. falciparum* isolates at the threshold of (a) BP_{TS} ≥0.50 to define clusters of genetically-related isolates based on the posterior mean BP_{TS} estimates. Every node represents a *P. falciparum* isolate and an edge represents the BP_{TS} value between two particular nodes/isolates. Isolates that cluster together (i.e., connected by edges) are considered to be genetically identical (i.e., clones). Every color represents a different *var*code. The (b) number and (c) proportion of pairwise comparisons within a given 95% highest density posterior interval width, which provides a measure of the uncertainty of each pairwise estimate. For the full distribution of within-*var*code BP_{TS} estimates see Fig. S5d-f.

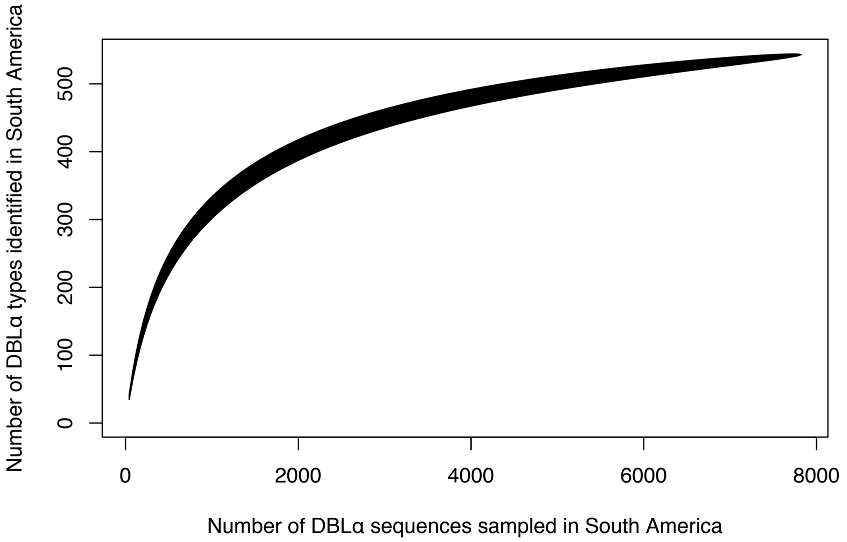

Figure S7. Sampling depth of *var* DBLα types in South America. Sampling accumulation curves depict the number of observed DBLα types plotted as a function of the number of DBLα sequences sampled.

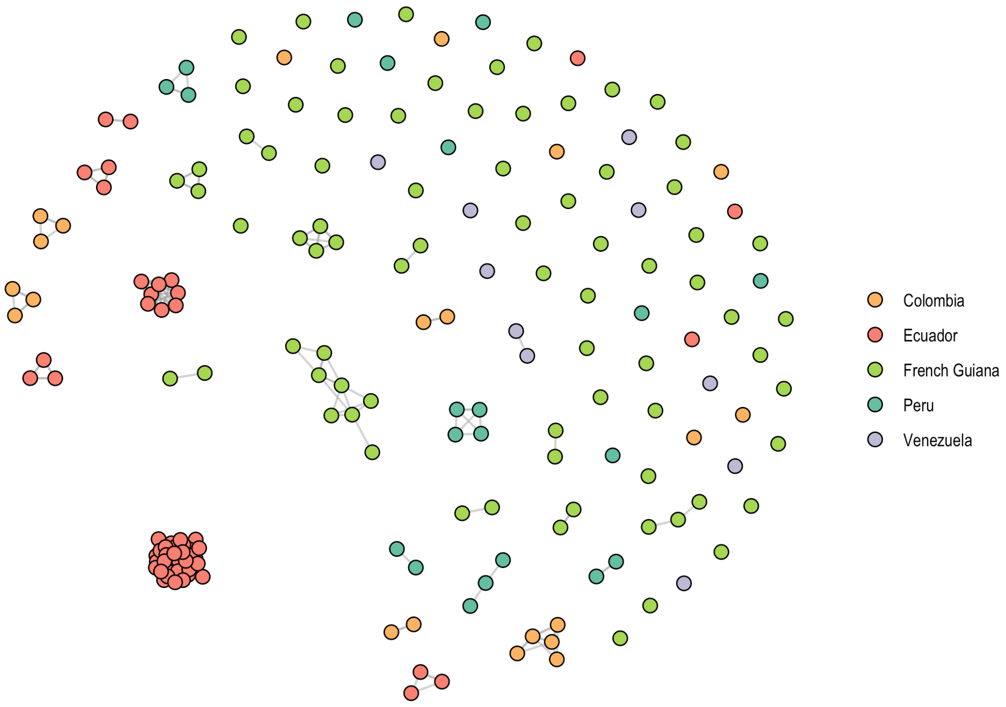

Figure S8. South American *var*codes. A network visualization of the *var*code relatedness of *P. falciparum* isolates at the threshold of P_{TS} ≥0.90 to define *var*codes in South America (see Methods). Every node represents a *P. falciparum* isolate and an edge represents the P_{TS} value between two particular nodes/isolates. Isolates that cluster together (i.e., connected by edges) are considered to have the same *var*code. The colors depict the country of origin/sampling location of each *P. falciparum* isolate.

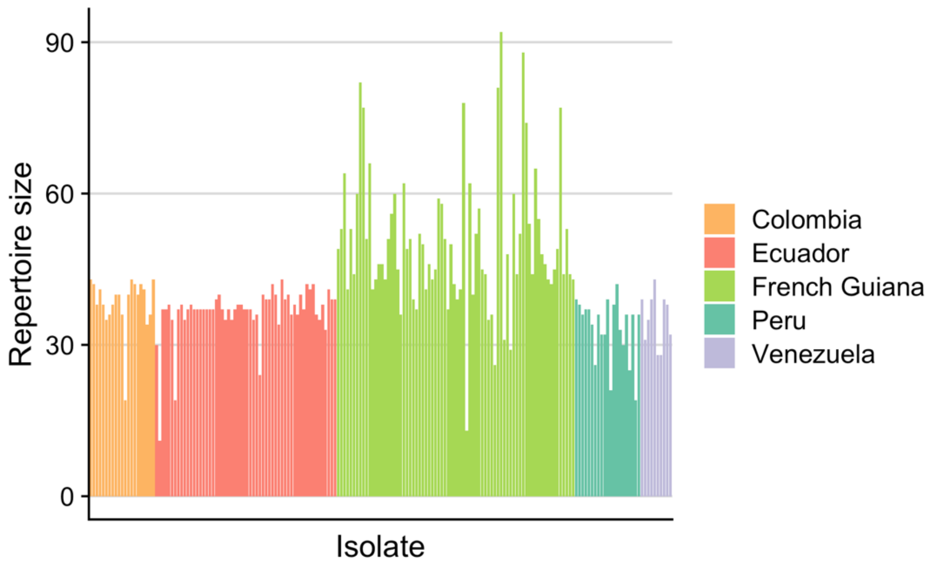

Figure S9. Repertoire sizes in South American *P. falciparum* isolates. The median repertoire size ranged from 36.5 in Venezuela to 48.0 types in French Guiana. The maximum repertoire size was 42 or 43 in all countries except French Guiana that appeared to have more multi-genome infections (max = 92, i.e. complexity of infection > 1).

Supplementary Tables

Table S1. Summary statistics for total number of *var* DBLα types per isolate (i.e., repertoire size) in South American *P. falciparum* populations.

Location	min*	med	mean	max
Colombia	19.0	40.0	38.4	43.0
Ecuador	11.0	37.0	36.6	43.0
French Guiana	13.0	48.0	50.5	92.0
Peru	19.0	36.0	33.4	42.0
Venezuela	28.0	36.5	35.2	43.0

**Note:* all isolates with <10 types were removed due to low sequencing quality.

Supplementary References

1. He Q, Pilosof S, Tiedje KE, Ruybal-Pesántez S, Artzy-Randrup Y, Baskerville EB, Day KP, Pascual M. Networks of genetic similarity reveal non-neutral processes shape strain structure in Plasmodium falciparum. *Nat Commun* (2018) 9:1817. doi: 10.1038/s41467-018-04219-3
